# Marked sex differences in all-cause mortality on antiretroviral therapy in low- and middle-income countries: a systematic review and meta-analysis

**DOI:** 10.7448/IAS.19.1.21106

**Published:** 2016-11-08

**Authors:** Sarah W Beckham, Chris Beyrer, Peter Luckow, Meg Doherty, Eyerusalem K Negussie, Stefan D Baral

**Affiliations:** 1 Department of Epidemiology, Johns Hopkins Bloomberg School of Public Health, Baltimore, MD, USA; 2 Department of HIV/AIDS, World Health Organization, Geneva, Switzerland

**Keywords:** treatment, retention, all-cause mortality, men, males, gender, HIV/AIDS, developing countries

## Abstract

**Introduction:**

While women and girls are disproportionately at risk of HIV acquisition, particularly in low- and middle-income countries (LMIC), globally men and women comprise similar proportions of people living with HIV who are eligible for antiretroviral therapy. However, men represent only approximately 41% of those receiving antiretroviral therapy globally. There has been limited study of men’s outcomes in treatment programmes, despite data suggesting that men living with HIV and engaged in treatment programmes have higher mortality rates. This systematic review (SR) and meta-analysis (MA) aims to assess differential all-cause mortality between men and women living with HIV and on antiretroviral therapy in LMIC.

**Methods:**

A SR was conducted through searching PubMed, Ovid Global Health and EMBASE for peer-reviewed, published observational studies reporting differential outcomes by sex of adults (≥15 years) living with HIV, in treatment programmes and on antiretroviral medications in LMIC. For studies reporting hazard ratios (HRs) of mortality by sex, quality assessment using Newcastle–Ottawa Scale (cohort studies) and an MA using a random-effects model (Stata 14.0) were conducted.

**Results:**

A total of 11,889 records were screened, and 6726 full-text articles were assessed for eligibility. There were 31 included studies in the final MA reporting 42 HRs, with a total sample size of 86,233 men and 117,719 women, and total time on antiretroviral therapy of 1555 months. The pooled hazard ratio (pHR) showed a 46% increased hazard of death for men while on antiretroviral treatment (1.35–1.59). Increased hazard was significant across geographic regions (sub-Saharan Africa: pHR 1.41 (1.28–1.56); Asia: 1.77 (1.42–2.21)) and persisted over time on treatment (≤12 months: 1.42 (1.21–1.67); 13–35 months: 1.48 (1.23–1.78); 36–59 months: 1.50 (1.18–1.91); 61 to 108 months: 1.49 (1.29–1.71)).

**Conclusions:**

Men living with HIV have consistently and significantly greater hazards of all-cause mortality compared with women while on antiretroviral therapy in LMIC. This effect persists over time on treatment. The clinical and population-level prevention benefits of antiretroviral therapy will only be realized if programmes can improve male engagement, diagnosis, earlier initiation of therapy, clinical outcomes and can support long-term adherence and retention.

## Introduction

In 2015, some 37 million people worldwide were living with HIV. The past decade has seen a dramatic scale-up in antiretroviral treatment (ART) in low- and middle-income countries (LMIC). Nearly 12 million people in LMIC were on ART in 2013, a 30-fold increase from the 400,000 people on treatment in 2003 [1–3]. Recent trials have clearly demonstrated both the clinical and preventive benefits of early and sustained treatment [4,5], yet 22 million men, women and children living with HIV remain untreated, and more than 2 million HIV-associated deaths are estimated to occur annually [6]. Undiagnosed, untreated or insufficiently treated HIV infection remains an enormous global health challenge, and one for which tailored interventions are likely to be required, including interventions relevant for adult men living with HIV.

In the aggregate of all LMIC, men and women comprise similar proportions of adults living with HIV who are eligible for ART (49 and 51%, respectively). However, adult men represent only 41% of those receiving ART [2]. This trend is observed in most regions of the world and is most evident in the WHO African Region where men make up 44% of those living with HIV but only 36% of those receiving ART [2].

Attention towards sex differences in the global HIV pandemic has predominantly focused on the vulnerabilities – behavioural, biomedical and structural [7] – experienced by women and girls. In contrast, there has been relatively limited investigation on why men are less likely to enrol and to be retained in ART programmes, and why they have had higher HIV-associated mortality in many reports [8]. Although there is growing and essential attention to men in research on vulnerable populations (who have sex with men, transgender populations and people who use drugs), there remains a policy and programme “blind spot” regarding men and HIV outcomes in sub-Saharan Africa [9] and globally. A number of studies have suggested that male enrolees have higher mortality rates than females in HIV treatment studies [10]. Differential treatment outcomes by sex need to be understood to better design and specify interventions for men living with HIV.

The aim of this review is to elucidate sex differentials in mortality between men and women living with HIV and on ART. This review, in particular, focuses on all-cause mortality as an important treatment outcome.

## Methods

A systematic review (SR) and meta-analysis (MA) were conducted to assess differences in all-cause mortality between adult men and women living with HIV and on ART in LMIC. We used the Cochrane Group approach [11], following PRISMA guidelines [12].

### Inclusion and exclusion criteria

#### Study design

Any observational study design.

#### Populations

Male and female adults (age ≥15 years); in LMIC (World Bank 2012 definition) [13].

#### Exposures

On or initiated ART at the beginning of follow-up.

#### Outcomes

All-cause mortality, with data disaggregated by sex, even if sex differences and mortality were not the primary outcome.

#### Publication

Published in peer-reviewed journals from 1 January 2008 to 13 December 2013.

#### Language

Any language.

#### Exclusion

Total sample size <50 men or <50 women; or article type was a cost-effectiveness study, modelling, grey literature or literature review.

### Search strategy

Electronic searches of PubMed, EMBASE and Ovid Global Health were performed on 5 December 2013. With the assistance of an information specialist, the following medical subject heading (MeSH) terms were developed along with the LMIC terms for the PubMed search query, and similar terms were used as keywords for EMBASE and Ovid Global Health: “Anti-Retroviral Agents” OR “Antiretroviral Therapy, Highly Active,” which were together cross-referenced with the keywords “HIV” OR “AIDS” OR “HIV Infections” along with NOT queries for “Pediatrics” and “Children.” See the Supplementary material for the complete PubMed search term.

### Study selection and management of results

**Figure 1:**
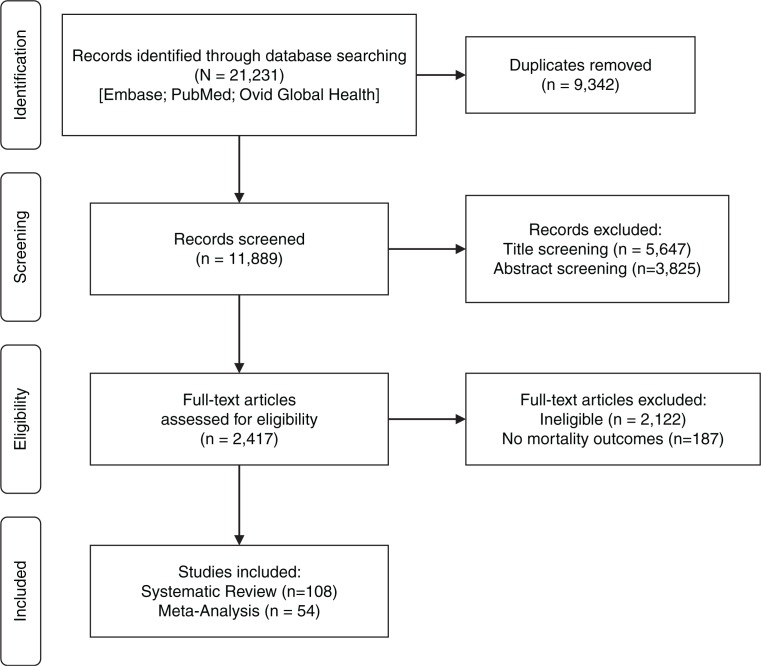
PRISMA flow diagram.

References obtained from three databases (*n*=21,231) were exported and duplicates were removed, leaving 11,889 citations. Reviewers and several research assistants independently conducted title screening (5604 records excluded), abstract screening (3825 excluded) and full-text review (6285 reviewed and 2352 excluded). Data were abstracted and collated through a Microsoft Access database that captured study information and mortality outcomes differentiated by sex. Title/abstract screening, full-text review and data abstraction were conducted in duplicate (in pairs by PL, SWB, KD, SP, EC, AG, WE and RM) and a third reviewer (SWB) resolved conflicts. This was conducted as part of a larger SR that abstracted data on multiple baseline factors (age, CD4 count and WHO stage) and outcomes (e.g. mortality, viral load (VL), CD4 count and opportunistic infections) (*n*=295 articles with such outcomes disaggregated by sex). For this analysis, all records with any mortality data disaggregated by sex were included (*n*=108 records) (see Figure 1).

### Statistical analyses

Using Stata 14.0 (College Station, TX, USA), a random-effects MA was conducted on reported hazard ratios (HRs) between men and women for all-cause mortality, using the *metan* command. Reciprocals of HRs were calculated as necessary such that all HRs used women as the reference group. *I*^*2*^ test statistics were calculated to assess heterogeneity, and an *a priori* cutoff of *I*^*2*^ values above 80% was chosen [11]. To assess the potential for publication bias, a funnel plot and *p*-value were generated using Stata (*metafunnel* and *metabias* commands with the Egger option and using standard error).

### Sensitivity and subgroup analyses

To address possible methodological and clinical heterogeneity [11], several decisions were made. Cohort and case–control studies are systematically different and not recommended to pool in MA; thus, case–control studies were excluded (*n*=1), as were cross-sectional studies (*n*=1). Studies reporting only crude mortality (*n*=17), odds ratios (*n*=8) or relative risk ratios (*n*=4) rather than HRs were likewise excluded [11]. Some studies reported HRs, but combined mortality with losses to follow-up in one measure, so were excluded from analysis (*n*=4). Articles were assessed for use of data from the same cohorts. Several potential overlaps were identified and the studies with larger sample sizes and longer follow-up times were retained, while 16 studies were dropped from the MA. See Supplementary Table 2 for a complete list of the original 108 studies, which were included in or excluded from the final MA, with reasons.

#### Subgroup analysis

Several subgroups were predetermined and reported separately (geography, time on ART and population characteristics).

##### Geography

The studies were grouped by geographic regions (sub-Saharan Africa (SSA), Asia, Latin American and the Caribbean (LAC), and studies that cover multiple regions). The one study from Eastern Europe/central Asia (Georgia) [14] was grouped with Asia. Pooled HRs (pHRs) were calculated overall and for each subgroup. Because there was only one study from LAC, it was not reported as a separate subgroup, but did contribute to the overall pooled effect size. Because there were a large number of studies from SSA, these were further subgrouped into geographic regions [15]: East (Ethiopia, Kenya, Malawi, Rwanda, Tanzania and Uganda); West/Central (Burkina Faso, Cameroon, Côte d’Ivoire, Gambia, Nigeria and Senegal); Southern (Botswana, Lesotho, Mozambique, South Africa, Zambia and Zimbabwe); and studies that cross multiple subregions (e.g. one study covering Central African Republic, Côte d’Ivoire, Democratic Republic of the Congo, Ethiopia and Niger).

##### Time on ART

Time on ART was divided into quartiles of months since initiation of ART (0–12, 13–35, 36–59 and 60–108 months). For studies that reported more than one HR with time overlaps (e.g. 0–3 and 0–12 months), the shorter time period was dropped. For studies that reported more than one HR without time overlaps (e.g. 0–12 and 13–24 months), all HRs were used.

##### Population characteristics

Certain features of the populations that may affect analyses and interpretation or increase heterogeneity were noted (e.g. tuberculosis co-infection, ART naïve or experienced and people who inject drugs (PWID)). The one study of tuberculosis co-infected patients [16] and one with the most immunosuppressed patients (CD4 count <50/mL) [17] were excluded. The proportion of HIV infected who were PWID were noted, when reported. Some mortality among PWID may be attributable to drug overdose, and men are more likely to inject drugs than women [18], thus possibly confounding the results. There were four studies in Asia where the proportion of reported PWID was ≥20%. These are subgrouped in MA and reported separately from the rest of the countries in Asia.

### Quality assessment

To assess the risk of bias, the Newcastle–Ottawa Quality Assessment Scale (NOS) was used [19]. This scale is used to assess the quality of observational studies in meta-analyses. For this article, the scale was used to rate all studies that reported HRs of mortality comparing men and women. As all studies reporting HRs were cohort studies, the NOS Coding Manual for Cohort Studies was employed. For each study, this scale addressed selection bias, comparability bias and outcome bias. Studies were awarded stars when they satisfied a requirement on the scale, with a maximum of nine stars. To calibrate scoring and minimize rating bias, two reviewers (including the first author) independently and dually applied the NOS to six randomly selected studies (10%). Once we aligned the scores, one reviewer applied the scale to the remaining studies, which were then checked by the first author. Studies that earned 1 to 3 stars were considered “low” quality; 4 to 6 stars “moderate”; and 7 to 9 stars were considered “high” quality.

The NOS requires that decisions be made to fit the particular analysis. The following decisions were discussed by authors and applied. For *selection*, (1) the exposure is sex, for example, males are exposed and females are non-exposed; (2) the community is people living with HIV and on ART. Thus, studies received a star for representativeness if the exposed cohort was a random sample of those in care and on ART, and another star if the females came from the same care centres as the males. Ascertainment of exposure (sex) received a star if the study drew this information from medical records. The NOS provides guidance for defining the outcome of interest in mortality studies and suggests using the presence of disease/incident, rather than death, as the outcome of interest. In this case, “known to be living with HIV” was used as the baseline disease and earned a star. As being on ART was an inclusion criterion, it is assumed that all were seropositive by biological measures (e.g. not self-reported living with HIV).

To assess *comparability* of cohorts, adjustment for factors that could be related to both the exposure (sex) and the outcome (all-cause mortality) were considered important. One star was awarded if the study controlled for age (first important factor) and a second star if the study also controlled for two or more variables from at least two distinct categories (see Supplementary Table 1 for a complete list of variables adjusted for in each study):

Category 1: CD4 count, VL and WHO stage

Category 2: Weight, body mass index (BMI) and mid-upper arm circumference (MUAC)

Category 3: Comorbidities (including previous or current tuberculosis)

Category 4: Adherence

Category 5: Risk behaviours (i.e. drug use)

To assess *outcome* bias, the method of assessing the outcome (mortality) earned a star if the information came from medical records and/or from confirmation by a health worker. The appropriateness of the follow-up time was defined as at least three months of follow-up. The adequacy of follow-up of cohorts was awarded a star if there was <15% loss to follow-up (LTFU); a higher percentage could be awarded a star if a description and comparison of those LTFU was included, for example, it was evaluated as an outcome.

To test for effects of study quality on pooled effect size, sensitivity analyses were run (see Table 4) on six models: (1) all studies that reported HRs of mortality, (2) only studies that earned a high-quality rating (7–9 stars), (3) only studies that had LTFU rates of <18% and (4) only studies that LTFU rates of <15%. Additional sensitivity analyses were run further on Model 4 to exclude studies that did not adjust for age (*n*=2) (Model 5) and exclude studies that were outliers in the funnel plot (see Supplementary Figure 1) and thus presented potential publication bias of extreme results (*n*=2) (Model 6).

## Results

There were 108 studies included in the SR of sex differences in mortality on ART in LMIC. The characteristics of the included studies are in Table 1. Most studies were from sub-Saharan Africa, were cohort studies of ART-naïve patients followed from initiation of ART and were among general population, reproductive age adults. The median follow-up time was 36 months (mean=42 months). The studies included a total of 319,677 men and 466,822 women.

**Table 1:** Characteristics of included studies

First author, year; country; study design^a^	Population (adults (age ≥15 years) on ART) and setting	Time^b^	Male (*n*)	Female (*n*)	References
Africa					
Abaasa, 2008; Uganda; R	ART naïve; urban clinic	31.2	222	675	[20]
Ahonkhai, 2012; South Africa; R	ART naïve; urban and rural clinics and hospitals	60	3818	7579	[21]
Alamo, 2012; Uganda; R	ART experienced and naïve; urban clinics and home-based care	120	237	342	[22]
Alemu, 2010; Ethiopia; R	ART naïve; rural hospital	24	117	155	[23]
Alibhai, 2010; Uganda; P	ART naïve; rural community- and hospital-based clinics	5.5	163	222	[24]
Balcha, 2010; Ethiopia; R	ART naïve; urban and rural clinics and hospitals	24	703	1006	[25]
Bastard, 2011; Senegal; P	ART naïve; urban hospital	108	146	184	[26]
Biadgilign, 2012; Ethiopia; R	ART naïve; hospitals	60	574	963	[27]
Birbeck, 2011; Zambia; P	ART naïve; rural clinics	24	205	291	[28]
Bisson, 2008; Botswana; R	ART naïve; urban clinic	12	166	244	[29]
Boyles, 2011; South Africa; P	ART naïve; rural hospital and clinics	48	563	1231	[30]
Brennan, 2013; South Africa; R	ART naïve, non-pregnant; urban clinic	48	5770	9162	[31]
Brinkhof, 2009, 4 countries^c^; R	ART naïve; urban clinics	24	4418	8831	[32]
Chalamilla, 2012; Tanzania; P	ART naïve; urban clinics	36	4383	8459	[33]
Chen, 2008; Malawi; R	ART naïve, urban hospital	30	1122	1716	[34]
Chi, 2009; Zambia; P	ART ≥12 months; urban clinics	36	10,226	16,889	[35]
Chi, 2010; Zambia; R	ART naïve; urban clinics	30	4618	5867	[36]
Chu, 2010; Malawi; R	ART naïve; rural hospital	13	4369	6753	[37]
Cornell, 2009; South Africa; P	ART naïve; urban clinic	12	717	1479	[38]
Cornell, 2012; South Africa; R	ART naïve; urban and rural clinics and hospitals	84	16,108	30,093	[39]
Dalal, 2008; South Africa; R	Patients LTFU ≥6 weeks; urban clinic	15	554	1077	[40]
De Beaudrap, 2008; Senegal; P	95% ART naïve, 5% experienced; urban multiple settings (ANRS-1215)	84	183	221	[41]
De Luca, 2012; Guinea, Malawi, Mozambique; P	ART naïve; urban and rural clinics	40	954	1558	[42]
Deribe, 2013; Ethiopia; CC	ART experienced, TB co-infected; urban hospital	48	124	149	[43]
Desilva, 2009; Nigeria; R	ART naïve; urban clinic	24	452	1100	[44]
Ekouevi, 2010; 5 countries^d^; P	ART naïve; urban clinics	12	5542	8810	[45]
Evans, 2012; South Africa; R	ART naïve; urban clinic	12	3205	5204	[46]
Fatti, 2010; South Africa; R	ART naïve urban and rural clinics and hospitals	36	9317	19,886	[47]
Ford, 2010; Lesotho; P	ART naïve; urban and rural clinics and hospitals	24	400	801	[48]
Fox, 2010; South Africa; R	ART naïve; urban clinic	48	2229	3976	[49]
Fox, 2012; South Africa; R	ART naïve, ≥6 months follow-up; urban/rural hospitals/clinics	96	6665	12,980	[50]
Franke, 2011; Rwanda; R	ART-naïve CD4 ≥350, with TB; urban/rural hospitals/clinics	24	121	187	[16]
Geng, 2010; Uganda; P	ART naïve, plus LTFU; rural clinic	45	1415	2213	[51]
Geng, 2010; Uganda; P	ART naïve, plus LTFU; rural clinic	45	1415	2213	[52]
Greig, 2012; 9 countries^e^; R	ART naïve; urban and rural clinics and hospitals	24	6201	11,360	[53]
Hawkins, 2011; Tanzania; P	ART naïve; urban clinics	33	4383	8459	[54]
Hermans, 2012; Uganda; R	ART naïve; urban university-based clinic	12	2730	4929	[55]
Hoffmann, 2010; South Africa; P	ART naïve; community- and workplace-based clinics	12	8696	5401	[56]
Hoffmann, 2011; South Africa; P	ART naïve with ≥4 years follow-up; national clinics	48	9605	5455	[57]
Johannessen, 2008; Tanzania; P	ART naïve; rural hospital	36	97	223	[58]
Karstaedt, 2012; South Africa; R	ART naïve; urban hospital	56	1030	1608	[59]
Kassa, 2012; Ethiopia; R	ART naïve; urban hospital	60	1737	2473	[60]
Kebebew, 2012; Ethiopia; R	ART naïve; military personnel; urban hospital	12	548	186	[61]
Kipp, 2012; Uganda; P	ART naïve; community- and hospital-based, urban and rural patients	24	163	222	[62]
Kouanda, 2012; Burkina Faso; R	ART naïve; urban and rural clinics	70	1682	3926	[63]
Lowrance, 2009; Rwanda; R	ART naïve; urban and rural clinics and hospitals	12	1123	2071	[64]
MacPherson, 2009; South Africa; R	ART naïve; rural clinics	24	446	907	[65]
Mageda, 2012; Tanzania; R	ART naïve; ≥12 months follow-up; urban and rural clinics and hospitals	60	226	320	[66]
Maman, 2012a; Malawi, Uganda, Kenya; R	ART ≥9 months and >1 CD4 count thereafter; urban/rural clinics	80	4068	8878	[67]
Maman, 2012b; Malawi, Uganda, Kenya; R	ART ≥9 months; urban/rural clinics	60	7682	16,355	[68]
Maman, 2012c; Malawi; P	ART naïve; rural clinic	12	235	338	[69]
Maskew, 2012; South Africa; R	ART naïve; urban clinic	24	3491	5648	[70]
Maskew, 2013; South Africa; R	ART naïve, non-pregnant; urban clinic	36	2733	4621	[71]
Massaquoi, 2009; Malawi; R	ART naïve; rural clinics/hospital	14	1449	2625	[72]
Moore, 2011; Uganda; P	ART naïve in home-based care; rural clinic/home-based care	60	306	826	[73]
Mossdorf, 2011; Tanzania; P	ART naïve; rural hospital; 7.6% with TB	12	518	945	[74]
Mosha, 2013; Tanzania; P	ART naïve; urban hospital	12	70	164	[75]
Mujugira, 2009; Botswana; R	ART naïve, CD4 <50; urban hospital	12	144	205	[17]
Murphy, 2010; South Africa; P	ART experienced patients with virologic failure; urban clinics	5.5	70	71	[76]
Mutevedzi, 2010; South Africa; P	ART naïve; urban/rural hospital/clinics	12	1836	3883	[77]
Mutevedzi, 2011; South Africa; R	ART naïve; urban/rural hospital/clinics	24	3011	5835	[78]
Mzileni, 2008; South Africa; P	ART naïve; urban hospital	18	1002	2071	[79]
Negin, 2011; Malawi; R	ART naïve; ≥25 years old; mixed settings	60	4099	6789	[80]
Nglazi, 2011; South Africa; P	ART naïve; semi-urban clinics	84	1046	2116	[81]
Odafe, 2012; Nigeria; R	ART naïve; non-pregnant; hospitals	36	1945	2840	[82]
Ojikutu, 2008; South Africa; R	ART naïve and experienced; urban clinic	62	132	174	[83]
Palombi, 2009; Guinea, Malawi, Mozambique; R	ART naïve; urban clinics	42	1800	2325	[84]
Palombi, 2010; Mozambique; P	ART naïve with ≥2 CD4 counts; urban and rural clinics	3	312	441	[85]
Peltzer, 2011; South Africa; P	ART naïve; urban and rural hospitals	12	217	518	[86]
Peterson, 2011, Gambia; P	ART naïve; urban hospital	36	121	238	[87]
Poka-Mayap, 2013; Cameroon; R	ART naïve; urban clinic	60	617	827	[88]
Rougemont, 2009; Cameroon; P	ART naïve; urban hospital	6	106	198	[89]
Russell, 2010; South Africa; P	ART naïve; urban clinics	9	542	808	[90]
Schoni-Affolter, 2011; Zambia; P	ART naïve; multiple sites; these data restricted to Zambia cohort	41	34,907	54,432	[91]
Sieleunou, 2009; Cameroon; R	ART naïve; rural hospital	60	660	527	[92]
Siika, 2010; Kenya; CC	ART experienced; randomly selected deceased patients matched 1:2 to living; urban/rural clinics/hospitals	69	613	968	[93]
Somi, 2012; Tanzania; R	ART naïve; national clinics	60	29,869	59,006	[94]
Steele, 2011; Botswana; P	ART naïve; urban clinic	6	152	250	[95]
Sunpath, 2012; South Africa; P	ART naïve patients with TB or other OIs; urban hospital	5.5	198	184	[96]
Taylor-Smith, 2010; Malawi; R	ART experienced, some naïve; HCWs, teachers, policy/army personnel; urban/rural hospitals/clinics	36	2346	2324	[97]
Toure, 2008; Côte d’Ivoire; P	ART naïve; mixed sites	32	3024	7187	[98]
Van Cutsem, 2011; South Africa; P	ART naïve; semi-urban clinics	24	2067	4344	[99]
Wandeler, 2012; Lesotho, Mozambique, Zimbabwe; P	ART ≥3 months; rural hospitals and clinics	36	2707	5018	[100]
Weigel, 2011; Malawi; CS	Patients LTFU ≥2 weeks; urban clinic	na	308	351	[101]
Wubshet, 2012; Ethiopia; R	ART naïve, non-pregnant; urban hospital	66	1349	1663	[102]
Zachariah, 2009; Malawi; R	ART naïve; rural clinics	3	706	1610	[103]
Eastern Europe/central Asia					
Tsertsvadze, 2011; Georgia; R	ART naïve; 60% drug users; national mixed settings	60	570	182	[14]
Latin American and Caribbean countries					
Wolff, 2010; Chile; P	ART naïve; transmission 2% drug use, >50% homosexual; mixed sites	84	4297	818	[104]
Asia					
Alvarez-Uria, 2013; India; R	ART experience unclear; drug use NR; rural hospitals	60	1876	1283	[105]
Argemi, 2012; Cambodia; R	ART naïve; drug use NR; rural clinic	56	498	504	[106]
Bastard, 2013; Laos; R	ART naïve; non-pregnant; urban hospital; drug use NR	60	507	405	[107]
Bhowmik, 2012; India; R	ART naïve; drug use NR; urban tertiary hospital	12	502	254	[108]
Chen, 2013; China; R	ART ≥3 months, 40% infected through drug use; rural/semirural clinics	14	1211	756	[109]
Dou, 2011; China; R	ART naïve, 20% infected through drug use; national ART database	24	2047	1410	[110]
Fregonese, 2012; Thailand; P	ART ≥3 months, past-PMTCT included; drug use NR; urban/rural hospitals	60	408	1170	[111]
Kumarasamy, 2008; India; P	ART ≥12 months; drug use NR; urban tertiary HIV centre	12	1512	460	[112]
Limmahakhun, 2012; Thailand; R	ART naïve and experienced with TB; drug use NR; urban hospital	120	100	71	[113]
Rai, 2013; India; R	ART naïve; drug use NR; urban clinic	36	182	57	[114]
Sabapathy, 2012; Burma; P	ART naïve; drug use NR; rural clinics	60	3656	2307	[115]
Susaengrat, 2011; Thailand; R	ART naïve; 88% heterosexual transmission (drug use NR); urban hospital	45	548	438	[116]
Thai, 2009, Cambodia; P	ART naïve; drug use NR; urban hospital	57	824	843	[117]
Tran, 2013; Vietnam; P	ART naïve; 65% infected through drug use; urban and rural clinics	6	2573	867	[118]
van Griensven, 2011; Cambodia; R	ART naïve; drug use NR; urban tertiary hospital	60	1333	1507	[119]
Zhang, 2008; China; R	ART naïve former plasma donors; data here are restricted to those on ART; mixed sites, national database	144	1302	1402	[120]
Zhang, 2009; China; P	ART naïve; 37.8% history of drug use; national database	60	28,320	20,441	[121]
Zhang, 2012; China; R	ART ≥7.5 months; 22% infected through drug use; national database	42	16,821	10,669	[122]
Multi-region studies					
Brinkhof, 2008; 11 countries^f^; P	ART naïve, drug use NR; multiple sites	12	2972	2519	[123]
Wandel, 2008; 3 countries^g^; R	ART naive; drug use NR; multiple sites	330	1272	800	[124]

ART, antiretroviral therapy; NR, not reported; na, not applicable; TB, tuberculosis; CD4, CD4+ cells/mL; OIs, opportunistic infections; LTFU, lost to follow-up; HCW, healthcare worker; PMTCT, prevention of mother-to-child transmission

a study design: P, prospective cohort; R, retrospective cohort; CS, cross-sectional; CC, case–control

b time in months since initiation of ART

c Côte d’Ivoire, Malawi, South Africa, Zimbabwe

d Benin, Côte d’Ivoire, Gambia, Mali, Senegal

e Central African Republic, Côte d’Ivoire, Democratic Republic of the Congo, Ethiopia, Nigeria, Republic of Congo, Uganda, Zambia, Zimbabwe

f Morocco, Botswana, Malawi, South Africa, Kenya, Côte d’Ivoire, Nigeria, Senegal, Brazil, India, Thailand

g Uganda, Côte d’Ivoire, Thailand.

Table 2 shows the mortality outcomes reported by the individual studies. Some studies reported only crude mortality, while others reported mortality per person-year and a majority reported a mortality ratio (odds ratio, relative risk or HR) comparing men and women. Significant findings are indicated in bold. Without exception, significant findings show worse mortality outcomes for men compared with women.

**Table 2:** All-cause mortality among adults on ART by sex

	Crude n (%)		Rate/100 py		cHR (95% CI), females		aHR (95% CI), females		
					
First author, date, country [Ref.]	Males	Females	Males	Females	Males	Females	Males	Females	Time^b^
Africa									
Abaasa, 2008, Uganda [20]	34 (15.3)	130 (19.3)	10.2	12.6	1.26 (0.86–1.83)	Ref	1.48 (0.98–2.17)	Ref	31.2
Ahonkhai, 2012, South Africa [21]	372 (9.7)	616 (8.1)	–	–	Ref	**OR 0.86 (0.75**–**0.99)**	–	–	6.9
Alamo, 2012, Uganda [22]	29 (12.2)	37 (10.8)	–	–	–	–	–	–	10
Alemu, 2010, Ethiopia [23]	–	–	–	–	1.22 (0.58–2.56)	Ref	–	–	24
Alibhai, 2010, Uganda [24]	22 (13.5)	20 (9.0)	–	–	–	–	–	–	5.6
Balcha, 2010, Ethiopia [25]	78 (11.1)	97 (9.6)	–	–	–	–	–	–	24
Bastard, 2011, Senegal [26]	–	–	–	–	–	–	Ref	**0.47 (0.26**–**0.84)**	108
Biadgilign, 2012, Ethiopia [27]	–	–	–	–	Ref	1.05 (0.68–1.64)	Ref	1.16 (0.68–2.00)	60
Birbeck, 2011, Zambia [28]	36 (17.6)	65 (22.5)	–	–	OR 0.72 (0.47–1.15)	Ref	–	–	24
Bisson, 2008, Botswana [29]	**36 (21.7)**, ***p*** **=0.03**	**33 (15.0)**	–	–	–	–	**1.74 (1.05**–**2.87)**^c^	Ref	12
Boyles, 2011, South Africa [30]	–	–	–	–	–	–	Ref	1.20 (0.87–1.66)	48
Brennan, 2013, South Africa [31]	906 (9.9)	1079 (18.7)	–	–	**RR 1.40 (1.30**–**1.60)**	Ref	RR 1.0 (0.90–1.10)	Ref	84
Brinkhof, 2009, 4 countries [32]	–	–	10.24 (9.66)^d^	6.98 (6.69)^d^	–	–	Ref	**0.84 (0.71**–**0.99)**	24
Chalamilla, 2012, Tanzania [33]	–	–	–	–	–	–	**1.19 (1.06**–**1.34)**, ***p*** **<0.001** **1.19 (1.07**–**1.32)**, ***p*** **<0.001**	Ref Ref	<3 36
Chen, 2008, Malawi [34]	**188 (16.8)**, ***p*** **<0.0001**	**188 (11.0)**	–	–	–	–	**1.70 (1.35**–**2.15)**	Ref	30
Chi, 2009, Zambia [35]	–	–	–	–	**1.40 (1.20**–**1.60)**	Ref	**1.30 (1.10**–**1.50)**	Ref	12–36
Chi, 2010, Zambia [36]	–	–	–	–	–	–	**1.82 (1.47**–**2.26)**	Ref	30
Chu, 2010, Malawi [37]	175 (0.13), KS	77 (22.3), KS	–	–	0.98 (0.73–1.31), KS	Ref	–	–	13
Cornell, 2009, South Africa [38]	51 (7.0) 14 (3.0)	59 (4.0) 32 (3.0)	**22.8**, ***p*** **=0.002** 3.5	**12.5** 3.8	**1.83 (1.26**–**2.26)**, ***p*** **=0.02** 0.98 (0.52–1.84)	Ref Ref	1.46 (0.96–2.22) 0.70 (0.37–1.34)	Ref Ref	0–4 4–12
Cornell, 2012, South Africa [39]	–	–	–	–	**1.28 (1.09**–**1.51)** **1.63 (1.37**–**1.94)** **1.62 (1.22**–**2.14)** **1.71 (1.08**–**2.70)** **1.46 (1.37**–**1.56)**	Ref Ref Ref Ref Ref	1.10 (0.93–1.31) **1.36 (1.05**–**1.78)** 1.39 (0.94–2.06) 1.35 (0.76–2.38**)** **1.31 (1.22**–**1.41)**	Ref Ref Ref Ref Ref	0–12 12–24 24–36 36–84 0–84
Dalal, 2008, South Africa [40]	31 (5.6)^c^	52 (4.8)^c^	–	–	–	–	–	–	15
De Beaudrap, 2008, Senegal [41]	–	–	–	–	Ref	1.03 (0.50–2.15) 0.69 (0.42–1.16)	–	–	0–6 6–84
De Luca, 2012, 3 countries [42]	–	–	–	–	Ref	**0.60 (0.40**–**0.90)**, ***p*** **=0.015**	Ref	**0.57 (0.36**–**0.90)**, ***p*** **=0.017**	6–48
Deribe, 2013, Ethiopia [43]	40 (32.3), TB	28 (18.8), TB	–	–	**OR 2.06 (1.18**–**3.59)**, TB	Ref	**OR 2.04 (1.04**–**4.02)**, ***p*** **=0.039**, TB	Ref	48
Desilva, 2009, Nigeria [44]	–	–	–	–	–	–	**1.76 (1.14**–**2.72)**, ***p*** **=0.0114**	Ref	24
Ekouevi, 2010, 5 countries [45]					**1.14 (1.08**–**1.21)**, ***p*** **=0.0004**^e^	Ref	**1.16 (1.10**–**1.24)**, ***p*** **=0.0002**^e^	Ref	12
Evans, 2012, South Africa [46]	–	–	–	–	1.17 (0.99–1.37)	Ref	–	–	12
Fatti, 2010, South Africa [47]	–	–	–	–	–	–	**1.14 (1.00**–**1.30)**, ***p*** **=0.047**	Ref	36
Ford, 2010, Lesotho [48]	–	–	–	–	–	–	0.90 (0.34–2.38)	Ref	24
Fox, 2010, South Africa [49]	278 (12.5)	398 (10.0)	–	–	**1.25 (1.08**–**1.44)**	Ref	–	–	48
Fox, 2012, South Africa [50]	–	–	3.34	3.46	Ref	1.02 (0.91–1.14)	Ref	0.95 (0.81–1.12); **0.86 (0.76**–**0.97)**^d^	6–96
Franke, 2011, Rwanda [16]	–	–	–	–	–	–	Ref	1.6 (0.8–3.2), TB	24
Geng, 2010a, Uganda [51]	46 (41.0)^f^	65 (59.0)^f^	–	–	0.85 (0.42–1.75)^f^	Ref	1.54 (0.53–4.12)^f^	Ref	45
Geng, 2010b, Uganda [52]	–	–	–	–	**2.19 (1.30**–**3.72)**, ***p*** **<0.01** 1.11 (0.65–1.92)^c^	Ref Ref	**1.86 (1.06**–**3.26)**, ***p*** **=0.03** 1.02 (0.57–1.83)^c^	Ref Ref	45
Greig, 2012, 9 countries [53]	–	–	–	–	**1.33 (1.14**–**1.54)**, ***p*** **<0.001** **1.34 (1.13**–**1.59)**, ***p*** **=0.001**	Ref Ref	**1.20 (1.01**–**1.41)**, ***p*** **=0.035** 1.02 (0.84–1.22)	Ref Ref	0–3 3–24
Hawkins, 2011, Tanzania [54]	643 (14.6)	1039 (12.3)	–	–	**1.23 (1.12**–**1.36)**, ***p*** **<0.01**	Ref	**1.19 (1.05**–**1.30)**, ***p*** **=0.001**	Ref	33
Hermans, 2012, Uganda [55]	–	–	–	–	**1.82 (1.47**–**2.25)**, ***p*** **<0.001**	Ref	**1.41 (1.12**–**1.77)**, ***p*** **=0.004**	Ref	12
Hoffmann, 2010, South Africa [56]	1044 (12.0)	432 (8.0)	–	–	Ref	**0.75 (0.66**–**0.86)**, ***p*** **<0.001**	Ref	0.89 (0.77–1.0)	12
Hoffmann, 2011, South Africa [57]	1706 (17.8)	952 (17.5)	9.85	9.03	**1.30 (1.20**–**1.50)** 1.20 (0.99–1.40) **1.30 (1.20**–**1.40)**, ***p*** **<0.001**	Ref Ref Ref	– – **1.20 (1.00**–**1.30)**, ***p*** **=0.004**	– – Ref	0–12 13–48 >48
Johannessen, 2008, Tanzania [58]	38 (39.2)	57 (25.6)	–	–	**1.73 (1.15**–**2.61)**, ***p*** **=0.009**	Ref	1.60 (1.00–2.57), *p*=0.053	Ref	36
Karstaedt, 2012, South Africa [59]	118 (11.5)	187 (11.6)	–	–	–	–	–	–	60
Kassa, 2012, Ethiopia [60]	–	–	–		1.19 (0.95–1.50)	Ref	1.07 (0.84–1.36)	Ref	60
Kebebew, 2012, Ethiopia [61]	64 (11.7)	22 (11.8)	–	–	–	–	–	–	48
Kipp, 2012, Uganda [62]	56 (31.8)^e^	58 (26.1)^e^	–	–	–	–	–	–	24
Kouanda, 2012, Burkina Faso [63]	–	–	–	–	**1.73 (1.49**–**2.02)**, ***p*** **<0.001**	Ref	**1.33 (1.05**–**1.68)**, ***p*** **=0.02**	Ref	70
Lowrance, 2009, Rwanda [64]	–	–	–	–	Ref Ref	**OR 0.67** **(0.46**–**0.98)** OR 0.88 (0.62–1.26)	Ref Ref	**OR 0.56** **(0.37**–**0.84)** 0.83 (0.56–1.12)	6 12
MacPherson, 2009, South Africa [65]	–	–	–	–	**1.55 (1.09**–**2.21)**	Ref	**1.63 (1.12**–**2.36)**	Ref	24
Mageda, 2012, Tanzania [66]	31 (13.7)	16 (5.0)	**7.83** **(5.51**–**11.14)**, ***p*** **=0.001**	**2.30** **(1.41**–**3.76)**	**3.19 (1.74**–**5.84)**	Ref	**4.71 (2.00**–**11.05)**, ***p*** **=0.001**	Ref	12–60
Maman, 2012a, 3 countries [67]	84 (2)	119 (1)	–	–	–	–	–	–	72
Maman, 2012b, 3 countries [68]	246	322	1.15	0.67	–	–	**1.33 (1.10**–**1.61)**	Ref	9–60
Maman, 2012c, Malawi [69]	60 (25.5)	58 (17.2)	85.3 (60/70.3 py)	55.6 (58/104.3 py)	**1.53 (1.07**–**2.19)**, ***p*** **=0.021**	Ref	1.36 (0.93–2.00)	Ref	12
Maskew, 2012, South Africa [70]	364 (10.4) 446 (12.8)	446 (7.9) 546 (9.7)	–	–	**1.35 (1.17**–**1.55)** **1.36 (1.20**–**1.54)**	Ref Ref	**1.23 (1.06**–**1.42)** **1.23 (1.08**–**1.41)**	Ref Ref	0–12 12–24
Maskew, 2013, South Africa [71]	143 (5.2)	190 (4.1)	–	–	1.00 (0.70–1.50) 1.20 (0.9–1.60) 1.30 (1.0–1.60)	Ref Ref Ref	1.00 (0.70–1.50) 1.20 (0.90–1.60) 1.20 (0.90–1.60)	Ref Ref Ref	0–12 12–24 24–36
Massaquoi, 2009, Malawi [72]	63 (4.3)	85 (3.2)	–	–	–	–	–	–	14
Moore, 2011, Uganda [73]	38 (10.8), *p*=0.093	74 (9.0)	–	–	–	–	–	–	60
Mossdorf, 2011, Tanzania [74]	130 (25.1)^e^	188 (19.9)^e^	–	–	Ref	**0.77 (0.62**–**0.97)** e	Ref	0.77 (0.52–1.15)^e^	12
Mosha, 2013, Tanzania [75]	14 (20.0)	21 (12.8)	–	–	–	–	–	–	12
Mujugira, 2009, Botswana [17]	–	–	–	–	Ref	0.83 (0.52–1.33) (CD4 <50)	Ref	0.68 (0.33–1.38) (CD4 <50)	12
Murphy, 2010, South Africa [76]	4 (6.0)	4 (6.0)	–	–	–	–	OR 1.50 (0.20–12.30)	Ref	5.3
Mutevedzi, 2010, South Africa [77]	–	–	–	–	**1.62 (1.49**–**1.77)**	Ref	**1.33 (1.06**–**1.67)**	Ref	12
Mutevedzi, 2011, South Africa [78]	–	–	–	–	–	–	**<50 yo, 1.64 (1.32**–**2.03)**	Ref	0–3
							**≥50 yo, 1.84 (1.06**–**3.17)**	Ref	0–3
							**<50 yo, 1.40 (1.09**–**1.80)**	Ref	3–12
							>50 yo, 1.33 (0.73–2.41)	Ref	3–12
							**1.95 (1.46**–**2.57)**	Ref	12–24
Mzileni, 2008, South Africa [79]	**193 (28.0)**, ***p*** **<0.0001**	**119 (8.0)**	–	–	–	–	–	–	18
Negin, 2011, Malawi [80]	–	–	–	–	–	–	Ref	**0.52 (0.46**–**0.60)**, ***p*** **<0.0001**	60
Nglazi, 2011, South Africa [81]	–	–	–	–	–	–	**1.48 (1.17**–**1.88)**, ***p*** **=0.001**	Ref	84
Odafe, 2012, Nigeria [82]	–	–	–	–	**1.70 (1.22**–**2.39)**, ***p*** **=0.002**	Ref	1.29 (0.89–1.86)	Ref	36
Ojikutu, 2008, South Africa [83]	26 (20.0)	23 (13.0)	–	–	Ref	0.69 (0.38–1.25)	Ref	0.75 (0.40–1.38)	62
Palombi, 2009, 3 countries [84]	–	–	–	–	Ref	**0.58 (0.48**–**0.71)**, ***p*** **<0.001**	Ref	**Model 1: 0.51 (0.40**–**0.63)**, ***p*** **<0.001** **Model 2: 0.45 (0.34**–**0.59)**, ***p*** **<0.001**	42
Palombi, 2010, Mozambique [85]	–	–	–	–	1.49 (0.96–2.31)	Ref	**1.81 (1.08**–**3.02)**	Ref	3
Peltzer, 2011, South Africa [86]	–	–	–	–	RR 1.21 (0.70–2.09)	Ref	–	–	12
Peterson, 2011, Gambia [87]	–	–	–	–	**2.00 (1.00**–**3.90)**, ***p*** **=0.0365** 2.20 (0.90–5.80)	Ref Ref	**4.90 (2.50**–**10.80)**, ***p*** **<0.0001** **2.50 (1.20**–**5.60)**, ***p*** **=0.0184**	Ref Ref	0–6 6–36
Poka–Mayap, 2013, Cameroon [88]	–	–	–	–	1.44 (0.94–2.11)	Ref	**2.15 (1.34**–**3.45)**, ***p*** **=0.002**	Ref	60
Rougemont, 2009, Cameroon [89]	–	–	–	–	Ref	OR 0.68 (0.30–1.55)	–	–	6
Russell, 2010, South Africa [90]	–	–	–	–	Ref	0.89 (0.65–1.23)	Ref	0.87 (0.63–1.20)	9
Schoni–Affolter, 2011, Zambia [91]	3986 (11.5)	4512 (8.3)	–	–	–	–	–	–	41
Sieleunou, 2009, Cameroon [92]	–	–	–	–	–	–	**1.73 (1.37**–**2.19)**, ***p*** **<0.001**	Ref	60
Siika, 2010, Kenya [93]	241 (39.3)	286 (29.5)	–	–	–	–	Ref	**0.71 (0.55**–**0.99)**	5.75
Somi, 2010, Tanzania [94]	3580 (12.0)	4746 (8.0)	–	–	Ref	**Min. estimate: 0.65 (0.62**–**0.68); Max. estimate: 0.95 (0.93**–**0.97)**	Ref	**Min. estimate 0.59 (0.56**–**0.62); Max. estimate: 0.88 (0.86**–**0.91)**	60
Steele, 2011, Botswana [95]	19 (12.5)	18 (7.2)	–	–	RR 1.74 (0.94–3.20)	Ref	–	–	6
Sunpath, 2012, South Africa [96]	49 (25.0)	49 (26.0)	–	–	0.90 (0.60–1.50)	Ref	–	–	5.5
Taylor–Smith, 2010, Malawi [97]	341 (14.5)	206 (8.8)	15.71 (14.13–17.47)	9.80 (7.93–10.41)	**RR 1.73 (1.45**–**2.06)**	Ref	**RR 1.90 (1.57**–**2.29)**	Ref	36
Toure, 2008, Côte d’Ivoire [98]	–	–	–	–	–	–	**1.52 (1.29**–**1.80)**, ***p*** **<0.0001**	Ref	32
Van Cutsem, 2011, South Africa [99]	–	–	–	–	**1.69 (1.16**–**2.47)**, ***p*** **=0.007** e	Ref	1.14 (0.74–1.76)^e^	Ref	24
Wandeler, 2012, 3 countries [100]	–	–	–	–	Ref	Sub-HR 0.69 (0.59–0.80), *p* <0.001	–	–	36
Weigel, 2011, Malawi [101]	–	–	–	–	–	–	1.0 (Ref)	OR 0.85 (0.56–1.29), *p*=0.45	na
Wubshet, 2012, Ethiopia [102]	–	–	–	–	–	–	**3.26 (2.19**–**4.88)**, ***p*** **<0.001**	Ref	66
Zachariah, 2009, Malawi [103]	84 (12.1), *p*=0.001	122 (7.7)	–	–	**OR 1.60 (1.20**–**2.20)**	Ref	**OR 1.60 (1.20**–**2.10)**, ***p*** **=0.03**	Ref	3
Eastern Europe/central Asia									
Tsertsvadze, 2011, Georgia [14]	–	–	–	–	**2.14 (1.38**–**2.32)**	Ref	**1.96 (1.19**–**3.24)**	Ref	60
Latin America/Caribbean									
Wolff, 2010, Chile [104]	–	–	–	–	1.23 (0.95–1.62)	Ref	0.81 (0.61–1.08)	Ref	84
Asia									
Alvarez-Uria, 2013, India [105]	–	–	–	–	–	–	Ref	**0.65 (0.52**–**0.83)**	60
Argemi, 2012, Cambodia [106]	–	–	–	–	Ref	0.78 (0.53–1.14)	–	–	56
Bastard, 2013, Laos [107]	–	–	–	–	–	–	Ref Ref	1.19 (0.69–2.05) **0.17 (0.07**–**0.44)**, ***p*** **<0.001**	0–9 10–60
Bhowmik, 2012, India [108]	43 (8.6)	13 (5.4)	–	–	–	–	–	–	12
Chen, 2013, China [109]	88 (7.3)		–	–	**2.70 (1.70**–**4.40)**	Ref	**2.10 (1.20**–**3.50)**	Ref	14
Dou, 2011, China [110]	292 (14.3)	151 (10.7)	–	–	**1.38 (1.13**–**1.68)** 1.12 (0.86–1.45) **1.56 (1.15**–**2.11)**	Ref Ref Ref	– 1.31 (0.95–1.81) **1.46 (1.04**–**2.06)**	– Ref Ref	24 <3 3–24
Fregonese, 2012, Thailand [111]	14 20	23 32	7.2 (4.2–12.1) 1.5 (1.0–2.3)	4.2 (2.8–6.3) 0.80 (0.6–1.2)	1.70 (0.90–3.40) **1.80 (1.00**–**3.01)**	Ref Ref	1.50 (0.70–3.10) **2.40 (1.20**–**4.80)**, ***p*** **=0.01**	Ref Ref	3–6 7–60
Kumarasamy, 2008, India [112]	**6.2%**, ***p*** **=0.033**	4.0%	–	–	–	–	–	–	12
Limmahakhun, 2012, Thailand [113]	4 (4.0), TB	2 (2.8), TB	–	–	–	–	–	–	120
Rai, 2013, India [114]	86 (47.3)	18 (31.6)	34.4 (27.9–42.5)	16.6 (10.4–26.3)	–	–	**2.80 (1.60**–**4.90)**	Ref	36
Sabapathy, 2012, Burma [115]	–	–	19.6 (17.6–21.8) 4.9 (4.4–5.5)	14.9 (12.8–17.3) 4.3 (3.7–4.9)	**1.31 (1.09**–**1.59)** 1.16 (0.87–1.38), *p*=0.09^e^	Ref Ref	1.29 (0.94–1.78) **1.63 (1.23**–**2.15)**, ***p*** **<0.001** e	Ref Ref	0–6 7–36
Susaengrat, 2011, Thailand [116]	75 (13.7)	50 (11.4)	–	–	–	–	–	–	45
Thai, 2009, Cambodia [117]	–	–	–	–	**1.55 (1.18**–**2.05)**, ***p*** **=0.002** e	Ref	**1.73 (1.29**–**2.32)**, ***p*** **<0.001** e	Ref	57
Tran, 2013, Vietnam [118]	141 (5.5)	57 (6.6)	–	–	Ref	**0.40 (0.29**–**0.57**)	Ref	**0.54 (0.34**–**0.85)**	0–6
Van Griensven, 2011, Cambodia [119]	60 (4.5) 38	37 (2.5) 26	10.45 1.27	5.44 0.77	Ref Ref	**0.52 (0.35**–**0.70)**, ***p*** **<0.01** **0.60 (0.36**–**0.99)**, ***p*** **=0.04**	Ref Ref	**0.48 (0.31**–**0.74)**, ***p*** **<0.01** **0.58 (0.34**–**0.99)**, ***p*** **=0.05**	0–6 6–60
Zhang, 2008, China [120]	208 (16)	144 (10.3)	–	–	**1.60 (1.30**–**2.00)**, ***p*** **<0.001**	Ref	**1.90 (1.20**–**2.90)**, ***p*** **=0.004**	Ref	144
Zhang, 2009, China [121]	**4136, (14.6)** ***p*****<0.001**	**2354 (11.5)**	9	5.7	**1.50 (1.40**–**1.50)**	Ref	**1.40 (1.03**–**1.50)**	Ref	60
Zhang, 2012, China [122]	–	–	–	–	**1.50 (1.40**–**1.70)**	Ref	**1.50 (1.20**–**1.70)**	Ref	6–48
Multi-regional									
Brinkhof, 2008, 11 countries [123]	–	–	–	–	–	–	Ref	0.83 (0.58–1.18)	6
Wandel, 2008, 3 countries [124]	–	–	–	–	Ref	Ranged 0.91–1.09, none significant	–	–	330

Bold indicates significant outcomes; cHR, crude, unadjusted hazard ratio; aHR, adjusted hazard ratio; CI, confidence interval; OR, odds ratios; RR, relative risk ratio; Ref, referent; sub-HR, subdistribution hazard ratio; TB, tuberculosis co-infection; py, person-years; KS, patients with Kaposi’s sarcoma; yo, years old

a Cox proportional hazard ratio, unless otherwise noted

b Time in months since initiation of ART

c Includes deaths among patients who were lost to follow-up and then traced (included in MA)

d with multiple imputations

e both known deaths and patients lost to follow-up (LTFU) in one measure (excluded from MA)

f exclusively patients who were LTFU and traced.

**Figure 2:**
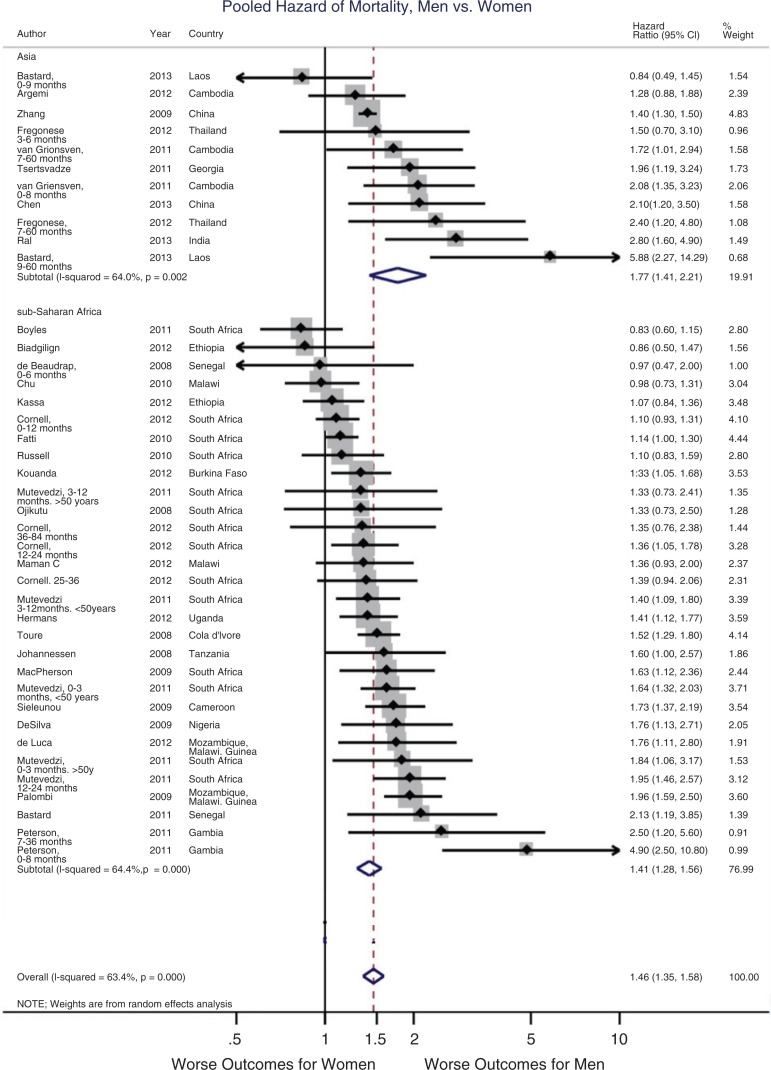
Forest plot of pooled hazard ratio of mortality on ART, men vs. women.

The results of an MA of HRs of mortality, comparing men with women, are presented in the forest plot in Figure 2 and Table 3. The final model of pHR of the included studies from LMIC was 1.46 (95% CI: 1.53–1.59), indicating men had a 46% increased hazard of death while on ART. The total sample size for this analysis was 203,952 (86,233 men and 117,719 women), and the total follow-up time was 1555 months (range=3–108 months). See Supplementary Table 2 for which studies that reported mortality were included and excluded and why.

**Table 3:** Pooled hazard ratios for mortality by sex and time on ART in lower- and middle-income countries

	Male (*n*)	Female (*n*)	≤12^a^	df	*I*^*2*^ (%)	13–35^a^	df	*I*^*2*^ (%)	36–59^a^	df	*I*^*2*^ (%)	60–108^a^	df	*I*^*2*^ (%)	Overall	df	*I*^*2*^ (%)
All LMIC	86,233	117,719	**1.42** **(1.21**–**1.67)** **p=0.002**	12	60.7	**1.48** **(1.23**–**1.78)** **p=0.027**	5	60.4	**1.50** **(1.18**–**1.91)** **p=0.000**	8	77.8	**1.49** **(1.29**–**1.71)** **p=0.005**	13	56.8	**1.46** **(1.35**–**1.59)** **p=0.000**	41	63.4

a Months since initiation of ART; df=degrees of freedom; Model 4.

After sensitivity analysis comparing six models (see Table 4), the final analysis included only studies with high-quality ratings on the NOS (7–9 stars) and had losses to follow-up of <15%. This resulted in 31 studies and 42 individual HRs in this analysis. The effect size of this final model compared with Model 1 (all 54 studies with 67 HRs, regardless of study quality and LTFU rates) is identical (1.46), but with a slightly wider confidence interval. However, the final model has a much lower *I* ^*2*^ (63.4% vs. 75.2%), indicating less heterogeneity between studies.

**Table 4:** Sensitivity analyses results

All LMIC	Male (*n*)	Female (*n*)	≤12^a^	df	*I* ^*2*^ (%)	13–35^a^	df	*I* ^*2*^ (%)	36–59^a^	df	*I* ^*2*^ (%)	60–108^a^	df	*I* ^*2*^ (%)	Overall	df	*I* ^*2*^ (%)
Model 1	186,452	283,811	**1.42 (1.25**–**1.63)** **p=0.002**	15	57.5	**1.39 (1.23**–**1.57)** **p=0.000**	13	70.1	**1.36 (1.22**–**1.52)** **p=0.000**	14	66.7	**1.62 (1.46**–**1.80)** **p=0.000**	21	74.8	**1.46 (1.38**–**1.56)** **p=0.000**	66	75.2
Model 2	175,554	266,621	**1.42 (1.25**–**1.63)** **p=0.002**	15	57.5	**1.40 (1.38**–**1.56)** **p=0.000**	12	72.3	**1.37 (1.22**–**1.53)** **p=0.000**	13	69.1	**1.62 (1.46**–**1.80)** **p=0.000**	21	74.8	**1.47 (1.38**–**1.56)** **p=0.000**	64	75.9
Model 3	119,470	168,465	**1.42 (1.25**–**1.63)** **p=0.002**	15	57.5	**1.43 (1.24**–**1.64)** **p=0.000**	11	70.8	**1.46 (1.21**–**1.75)** **p=0.000**	10	72.3	**1.49 (1.35**–**1.54)** **p=0.006**	14	54.2	**1.44 (1.35**–**1.54)** **p=0.000**	53	62.7
Model 4	86,233	117,719	**1.42 (1.21**–**1.67)** **p=0.002**	12	60.7	**1.48 (1.23**–**1.78)** **p=0.027**	5	60.4	**1.50 (1.18**–**1.91)** **p=0.000**	8	77.8	**1.49 (1.29**–**1.71)** **p=0.005**	13	56.8	**1.46 (1.35**–**1.59)** **p=0.000**	41	63.4
Model 5	85,552	116,994	**1.44 (1.22**–**1.71)** **p=0.000**	11	63.0	**1.48 (1.23**–**1.78)** **p=0.000**	5	60.4	**1.54 (1.18**–**2.02)** **p=0.002**	7	80.6	**1.49 (1.29**–**1.71)** **p=0.000**	13	56.8	**1.47 (1.35**–**1.60)** **p=0.000**	39	64.8
Model 6	85,431	116,756	**1.37 (1.20**–**1.56)** **p=0.000**	10	43.9	**1.48 (1.23**–**1.78)** **p=0.000**	5	60.4	**1.54 (1.18**–**2.02)** **p=0.002**	7	80.6	**1.43 (1.27**–**1.61)** **p=0.000**	12	42.2	**1.43 (1.33**–**1.55)** **p=0.000**	37	58.9

df, degrees of freedom; LTFU, lost to follow-up; HR, hazard ratio.

Model 1: all HRs.

Model 2: high-quality studies only (7–9 stars).

Model 3: HRs with LTFU <18%.

Model 4: HRs with LTFU <15% (all also scored high on the NOS).

Model 5: HRs with LTFU <15% and adjusted for age (excludes *n*=2 HRs).

Model 6: HRs with LTFU <15%, adjusted for age, and accounts for potential publication extremes on the funnel plot (excludes two additional HRs beyond Model 5).

a Months since initiation of ART

### Subgroup analyses

Analyses were run separately by geographic region, time since initiation of ART and injection drug use rates, including only studies eligible for the final model. Analyses were temporally stratified by quartiles of time since initiation of ART (0–12, 13–35, 36–59 and 60–108 months) (see Table 3). The overall significant effect of increased hazard of mortality for men persisted over time. In all LMIC, the pHR in the first year on ART was slightly ameliorated but still significant, showing a 42% increased hazard of death for men (95% CI: 1.21–1.67). This increased to 48% in months 13 to 35 (95% CI: 1.23–1.78), 50% in months 36 to 59 (95% CI: 1.18–1.91) and 49% in months 60 to 108 (95% CI: 1.29–1.71) since initiation.

For only SSA studies (pHR, *n*=30), the effect was slightly lower but still significant at 1.41 (1.28–1.56). In Asian countries (pHR, *n*=11), the effect was greater, with a 77% increased hazard of death for men (95% CI: 1.43–2.21, df=10, *I* ^*2*^=64.0%) (see Figure 2). pHRs for East, Southern and West/Central Africa are also calculated separately to explore differences by region given heterogeneity. The West/Central Africa subregion showed the worst outcomes, with all HRs above 70% higher for men (95% CI: 1.39–2.08, df=7, *I* ^*2*^=57.1%). East African studies showed a 19% increased hazard of death (95% CI: 1.01–1.41, df=5, *I* ^*2*^=36.7%), while Southern African studies showed a 33% increased hazard (95% CI: 1.18–1.51, df=13, *I* ^*2*^=58.1%).

Since the effect of the HR for mortality may be partly attributed to drug use deaths, particularly across Asian countries, we also calculated HRs for the studies that reported a proportion of their participants were PWID (reported between 20 and 60%). The pHR for studies with reported PWID was decreased (1.62 (95% CI: 1.23–2.14, df=1, *I* ^2^=47.3%)) compared with the overall effect, while the pHR for studies with no reported drug users was increased (1.85 (95% CI: 1.32–2.61, df=7, *I* ^*2*^=65.9%)).

### Quality assessment

Table 5 shows the results of risk of bias assessment using the NOS for observational cohort studies. Only studies that had the potential of being included in the final MA were assessed, for example, those that reported an HR for mortality. All but two studies scored a high rating (7–9 stars); lower scores generally reflected a lack of adjustment for key factors such as age and/or had high or unreported LTFU rates. Supplementary Table 1 indicates key baseline variables adjusted for in the mortality analyses: age; CD4 count, WHO stage and VL; weight (e.g. BMI); haemoglobin status; current or previous tuberculosis; ART start year and ART regimen; as well as other variables (listed in the table). In the final MA, only studies with <15% LTFU were included. All studies with <15% LTFU also earned high ratings.

**Table 5:** Quality assessment of studies included in the meta-analysis (Newcastle–Ottawa Scale for cohort studies)

	Selection				Comparability	Outcome			Total	Rating
			
Author, year [Reference]	Representativeness of the exposed cohort	Selection of the non-exposed cohort	Ascertainment of exposure	Demonstration that outcome of interest was not present at start of study	Comparability of cohorts on basis of design or analysis	Assessment of outcome	Was follow-up long enough for outcomes to occur	Adequacy of follow-up of cohorts	Number of stars (max. 9)	7–9=high; 4–6=moderate; 1–3=low
Africa										
Abaasa, 2008 [20]	*	*	*	*	*	*	*		7	High
Alemu, 2010 [23]	*	*	*	*		*	*		6	Moderate
Bastard, 2011 [26]	*	*	*	*	*	*	*	*	8	High
Biadgilign, 2012 [27]	*	*	*	*	**	*	*	*	9	High
Bisson, 2008 [29]	*	*	*	*	*	*	*	*	8	High
Boyles, 2011 [30]	*	*	*	*	**	*	*	*	9	High
Brinkhof, 2009 [32]	*	*	*	*	*	*	*		7	High
Chalamilla, 2012 [33]	*	*	*	*	**	*	*		8	High
Chen, 2008 [34]	*	*	*	*	**	*	*		8	High
Chi, 2009 [35]	*	*	*	*	**	*	*		8	High
Chi, 2010 [36]	*	*	*	*	**	*	*	*	9	High
Chu, 2010 [37]	*	*	*	*	*	*	*	*	8	High
Cornell, 2012 [39]	*	*	*	*	**	*	*	*	9	High
De Beaudrap, 2008 [41]	*	*	*	*		*	*	*	7	High
De Luca, 2012 [42]	*	*	*	*	**	*	*	*	9	High
Desilva, 2009 [44]	*	*	*	*	**	*	*	*	9	High
Fatti, 2010 [47]	*	*	*	*	*	*	*	*	8	High
Ford, 2010 [48]	*	*	*	*	*	*	*	*	8	High
Geng, 2010a [51]	*	*	*	*	*	*	*	*	8	High
Greig, 2012 [53]	*	*	*	*	**	*	*	*	9	High
Hawkins, 2011 [54]	*	*	*	*	**	*	*		8	High
Hermans, 2012 [55]	*	*	*	*	*	*	*	*	8	High
Hoffman, 2011 [57]	*	*	*	*	**	*	*		8	High
Johannessen, 2008 [58]	*	*	*	*	*	*	*	*	8	High
Kassa, 2012 [60]	*	*	*	*	**	*	*	*	9	High
Kouanda, 2012 [63]	*	*	*	*	**	*	*	*	9	High
MacPherson, 2009 [65]	*	*	*	*	**	*	*	*	9	High
Mageda, 2012 [66]	*	*	*	*	**	*	*		8	High
Maman, 2012b [68]	*	*	*	*	**	*	*	*	9	High
Maman, 2012c [69]	*	*	*	*	**	*	*		8	High
Mutevedzi, 2011 [78]	*	*	*	*	**	*	*	*	9	High
Negin, 2011 [80]	*	*	*	*	*	*	*		7	High
Odafe, 2012 [82]	*	*	*	*	*		*		6	Moderate
Ojikutu, 2008 [83]	*	*	*	*	**	*	*	*	9	High
Palombi, 2009 [84]	*	*	*	*	**	*	*	*	9	High
Peterson, 2011 [87]	*	*	*	*	*	*	*	*	8	High
Poka-Mayap, 2013 [88]	*	*	*	*	**	*	*		8	High
Russell, 2010 [90]	*	*	*	*	*	*	*	*	8	High
Sieleunou, 2009 [92]	*	*	*	*	*	*	*	*	8	High
Somi, 2012 [94]	*	*	*	*	**	*	*		8	High
Toure, 2008 [98]	*	*	*	*	**	*	*	*	9	High
Wandeler, 2012 [100]	*	*	*	*	*	*	*		7	High
Wubshet, 2012 [102]	*	*	*	*	*	*	*		7	High
Central Europe/East Europe										
Tsertsvadze, 2011 [14]	*	*	*	*	**	*	*	*	9	High
Latin America										
Wolff, 2010 [104]	*	*	*	*	**	*	*	*	9	High
Asia										
Alvarez-Uria, 2013 [105]	*	*	*	*	**	*	*		8	High
Argemi, 2012 [106]	*	*	*	*		*	*	*	7	High
Bastard, 2013 [107]	*	*	*	*	*	*	*	*	8	High
Chen, 2013 [109]	*	*	*	*	**	*	*	*	9	High
Fregonese, 2012 [111]	*	*	*	*	**	*	*	*	9	High
Rai, 2013 [114]	*	*	*	*	**	*	*	*	9	High
Tran, 2013, Vietnam [118]	*	*	*	*	**	*	*		8	High
Van Griensven, 2011 [119]	*	*	*	*	*	*	*	*	8	High
Zhang, 2009 [121]	*	*	*	*	*	*	*	*	8	High

*The study adequately met the criteria; 0–3 stars=low-quality rating, 4–6 moderate quality rating and 7–9 high-quality rating.

To assess the potential for publication bias, a funnel plot was generated and Egger’s test for small study effects was generated. A visual analysis of the funnel plot (see Supplementary Figure 1) indicates that there may be two extreme HRs. However, the confidence interval for Egger’s test overlaps the null, and the *p*-value is marginally insignificant (0.057), indicating no small study effects. A sensitivity analysis was conducted (see Table 4) excluding these two HRs (Model 6), which did not significantly change the outcome, however, so they were retained in the final (Model 4) analysis.

## Discussion

These analyses identified a consistent, significant and sustained sex differential in all-cause mortality between adult men and women living with HIV and on ART in LMIC. The data are consistent with previous studies suggesting higher mortality among men living with HIV on ART in sub-Saharan Africa [125]. However, the trend of increased mortality transcends SSA and is consistent across all LMIC with persistent sex disparities in mortality over time on treatment.

The differences between men’s and women’s mortality within the first 12 months are smaller, though still significant, showing worse outcomes for men as compared with women. For time on ART >12 months, the HRs retained significant and persisted over time. The sustained differential mortality suggests that even after the initial period on ART when there is typically a spike in mortality, there is a persistent and stronger hazard for men. This echoes data from South Africa, where the relationship between gender and increased mortality persists with increased duration on ART for those living with HIV [39]. Some of these effects may be attributable to baseline factors; men tended to be older when they initiated ART, for example, and presented at lower CD4 counts and higher VL in multiple reports [9,125,126]. Many of the studies in the MA adjusted for clinically relevant factors at baseline and ART initiation. Supplementary Table 1 shows which baseline factors were controlled for in all included studies, and adjusted HRs were used in the MA whenever they were available. Only two studies in the final model were not adjusted, and sensitivity analysis excluding those two did not change the results. Thus, baseline differences do not account for the differential mortality observed, though some confounding may remain and bias the results. Besides such baseline factors, the consistent and significant increased mortality among men can also be reflection of the higher background mortality rate from all other causes among men compared with women [39,127]. Men have been consistently shown to have higher mortality, which is multifactorial in nature, but in part related to limited access to or uptake of healthcare services. Since the men in this analysis were living with HIV and engaged in ART, there is an expectation that differential mortality is attenuated through engagement in care. In Cornell’s analysis from South Africa, the authors compared the increased hazard for mortality among men on ART (adjusted HR 1.31 (1.22–1.41)) to the background differential mortality and found that HIV-positive men in care were indeed protected from mortality – HIV-negative men had twice the hazard of death as men on ART [39]. This requires more investigation; despite being on treatment, men living with HIV are still dying significantly more than women.

These findings suggest that tailored interventions to improve early treatment initiation as well as treatment outcomes and reduce mortality on ART for men are urgently needed across Asia and Africa. Such interventions may likely be required across the HIV treatment cascade, but may be particularly important in several key domains. First, earlier diagnosis of HIV infection is likely to be critical, particularly given the individual clinical benefits of early initiation of ART as formally demonstrated in the START trial and currently recommended by WHO [4,128]. Since women, but not men, are much more frequent users of reproductive and other healthcare services, diagnoses and linkage to treatment may happen earlier especially through implementation of B+ programmes [129]. Improved outreach to men at risk will likely need to go beyond the clinic to where men work, socialize and engage in risk, and include risk-leveraging approaches such as HIV self-testing. In addition, clinic hours and wait times that are fit to the working hours and demands of men are likely critical, as are gender-transformative interventions [130]. The findings of durable differentials in mortality out to five years post-ART initiation call for greater understanding of underlying causes, including challenges related to treatment adherence and retention on ART for men specifically. There is relatively little research on adherence and retention differentials by sex [8,9], and throughout completion of this SR, randomized evaluations which reported treatment outcomes by sex were rarely found. There is also a need for greater attention to and interventions for conditions which tend to affect men more and exacerbate HIV/AIDS prevention and treatment, and increase men’s morbidity and mortality, such as tuberculosis [131] and substance use [18].

This study has several limitations. Due to the nature of the available data, the main outcome of these analyses was all-cause mortality, rather than HIV-related mortality. While HIV-related mortality would be a more precise outcome, these data were rarely reported in studies found in this review, likely due to limitations in mortality reporting in LMIC. Thus, these findings likely include deaths from causes other than HIV/AIDS that differentially affect men, such as substance use and intentional and unintentional violence, as well as AIDS-related causes such as tuberculosis. Furthermore, only a small proportion of outcome data found in the SR was disaggregated by sex; hence, this review was limited to those that completed stratified analyses and/or reported sex-stratified data.

An additional caution in LMIC settings is the paucity of data on treatment outcomes among patients who are LTFU. In several settings, this group includes unascertained death, self-referral to other clinics and care discontinuation or “true lost to follow ups.” To address this issue, the sensitivity analysis ran multiple models allowing for different LTFU rates (all studies, LTFU <18% only and LTFU <15% only), and the final reported pHR includes only studies with <15% LTFU. However, there was no difference in the pHRs between the full and restricted models (both 1.46 with only a slightly widened 95% CI in the restricted model; see Table 4). This review did not include studies that exclusively reported on men’s treatment outcomes, which may give more insight into specific treatment outcomes. In particular, significant data on predominantly male populations living with HIV would be harnessed in studies on vulnerable and key populations including men who have sex with men (MSM) and men who inject drugs. The eligibility criterion of including both men and women necessarily excluded those studies if they did not compare men’s and women’s outcomes. Interventions for these populations may provide insight into interventions to better tailor services for men living with HIV whose acquisition risks have not been defined as well. Finally, this review also only included quantitative outcomes. Qualitative studies would illuminate some of the reasons for these sex differentials, providing testable hypotheses and representing an important next step in informing interventions to address this disparity.

Taken together, these findings call for greater attention to sex and gender as a factor in the analyses of outcomes, given the importance of sex as a determinant of mortality reported here. It is also important to understand how much of the differential mortality among people living with HIV and on ART is due to background mortality, for example, a decreased life expectancy of men in the general population. Additionally, we could not assess risk factors for HIV acquisition, largely because such data are rarely collected in treatment programmes. However, some proportion of adult men across sub-Saharan Africa and Asia also belong to stigmatized and harder to reach subgroups, including PWID, MSM, male sex workers, prisoners, men in uniform, and transnational migrants and seasonal and migrant workers. All of these groups may face additional determinants of risk including lower access to health services, greater likelihood of treatment interruptions, discrimination in healthcare services and other social and structural barriers to continuity of treatment [132,133]. An implementation research agenda is called for to assess the optimal strategies to link and retain in treatment these men who are living with HIV, given additional stigma that can exclude or cause men to self-exclude from diagnostic and treatment services.

## Conclusions

Consistent differentials in HIV outcomes for men pose an additional challenge: control of HIV incidence among their sexual partners. As long as men living with HIV are significantly less likely to be virally suppressed, suboptimal clinical outcomes will manifest, combined with ongoing risks of onward HIV transmission to all within their sexual networks. This is perhaps most true across SSA, where sexual transmission of HIV predominates and improved HIV prevention for women and girls is vital. To realize HIV prevention gains, the higher proportion of untreated men in these settings must be addressed. In short, improving HIV clinical outcomes for men is an urgent public health priority.

## Supplementary Material

Marked sex differences in all-cause mortality on antiretroviral therapy in low- and middle-income countries: a systematic review and meta-analysis
