# Supplementary material for: Marked sex differences in all-cause mortality on antiretroviral therapy in low- and middle-income countries: a systematic review and meta-analysis
Source: J Int AIDS Soc. 2016 Nov 8;19(1):21106. doi: 10.7448/IAS.19.1.21106 (PMC5103676; doi:10.7448/IAS.19.1.21106)

Appendix

PubMed Search Query

((((((((("developing country"[tiab] OR "developing countries"[tiab] OR "developing nation"[tiab] OR "developing nations"[tiab] OR "developing population"[tiab] OR "developing populations"[tiab] OR "developing world"[tiab] OR "less developed country"[tiab] OR "less developed countries"[tiab] OR "less developed nation"[tiab] OR "less developed nations"[tiab] OR "less developed population"[tiab] OR "less developed populations"[tiab] OR "less developed world"[tiab] OR "lesser developed country"[tiab] OR "lesser developed countries"[tiab] OR "lesser developed nation"[tiab] OR "lesser developed nations"[tiab] OR "lesser developed population"[tiab] OR "lesser developed populations"[tiab] OR "lesser developed world"[tiab] OR "under developed country"[tiab] OR "under developed countries"[tiab] OR "under developed nation"[tiab] OR "under developed nations"[tiab] OR "under developed population"[tiab] OR "under developed populations"[tiab] OR "under developed world"[tiab] OR "underdeveloped country"[tiab] OR "underdeveloped countries"[tiab] OR "underdeveloped nation"[tiab] OR "underdeveloped nations"[tiab] OR "underdeveloped population"[tiab] OR "underdeveloped populations"[tiab] OR "underdeveloped world"[tiab] OR "middle income country"[tiab] OR "middle income countries"[tiab] OR "middle income nation"[tiab] OR "middle income nations"[tiab] OR "middle income population"[tiab] OR "middle income populations"[tiab] OR "low income country"[tiab] OR "low income countries"[tiab] OR "low income nation"[tiab] OR "low income nations"[tiab] OR "low income population"[tiab] OR "low income populations"[tiab] OR "lower income country"[tiab] OR "lower income countries"[tiab] OR "lower income nation"[tiab] OR "lower income nations"[tiab] OR "lower income population"[tiab] OR "lower income populations"[tiab] OR "underserved country"[tiab] OR "underserved countries"[tiab] OR "underserved nation"[tiab] OR "underserved nations"[tiab] OR "underserved population"[tiab] OR "underserved populations"[tiab] OR "underserved world"[tiab] OR "under served country"[tiab] OR "under served countries"[tiab] OR "under served nation"[tiab] OR "under served nations"[tiab] OR "under served population"[tiab] OR "under served populations"[tiab] OR "under served world"[tiab] OR "deprived country"[tiab] OR "deprived countries"[tiab] OR "deprived nation"[tiab] OR "deprived nations"[tiab] OR "deprived population"[tiab] OR "deprived populations"[tiab] OR "deprived world"[tiab])) OR ("poor country"[tiab] OR "poor countries"[tiab] OR "poor nation"[tiab] OR "poor nations"[tiab] OR "poor population"[tiab] OR "poor populations"[tiab] OR "poor world"[tiab] OR "poorer country"[tiab] OR "poorer countries"[tiab] OR "poorer nation"[tiab] OR "poorer nations"[tiab] OR "poorer population"[tiab] OR "poorer populations"[tiab] OR "poorer world"[tiab] OR "developing economy"[tiab] OR "developing economies"[tiab] OR "less developed economy"[tiab] OR "less developed economies"[tiab] OR "lesser developed economy"[tiab] OR "lesser developed economies"[tiab] OR "under developed economy"[tiab] OR "under developed economies"[tiab] OR "underdeveloped economy"[tiab] OR "underdeveloped economies"[tiab] OR "middle income economy"[tiab] OR "middle income economies"[tiab] OR "low income economy"[tiab] OR "low income economies"[tiab] OR "lower income economy"[tiab] OR "lower income economies"[tiab] OR "low gdp"[tiab] OR "low gnp"[tiab] OR "low gross domestic"[tiab] OR "low gross national"[tiab] OR "lower gdp"[tiab] OR "lower gnp"[tiab] OR "lower gross domestic"[tiab] OR "lower gross national"[tiab] OR lmic[tiab] OR lmics[tiab] OR "third world"[tiab] OR "lami country"[tiab] OR "lami countries"[tiab] OR "transitional country"[tiab] OR "transitional countries"[tiab])) OR (Africa[tiab] OR Asia[tiab] OR Caribbean[tiab] OR West Indies[tiab] OR South America[tiab] OR Latin America[tiab] OR Central America[tiab] OR "Atlantic Islands"[tiab] OR "Commonwealth of Independent States"[tiab] OR "Pacific Islands"[tiab] OR "Indian Ocean Islands"[tiab] OR "Eastern Europe"[tiab] OR Afghanistan[tiab] OR Albania[tiab] OR Algeria[tiab] OR Angola[tiab] OR Antigua[tiab] OR Barbuda[tiab] OR Argentina[tiab] OR Armenia[tiab] OR Armenian[tiab] OR Aruba[tiab] OR Azerbaijan[tiab] OR Bahrain[tiab] OR Bangladesh[tiab] OR Barbados[tiab] OR Benin[tiab] OR Byelarus[tiab] OR Byelorussian[tiab] OR Belarus[tiab] OR Belorussian[tiab] OR Belorussia[tiab] OR Belize[tiab] OR Bhutan[tiab] OR Bolivia[tiab] OR Bosnia[tiab] OR Herzegovina[tiab] OR Hercegovina[tiab] OR Botswana[tiab] OR Brasil[tiab] OR Brazil[tiab] OR Bulgaria[tiab] OR Burkina Faso[tiab] OR Burkina Fasso[tiab] OR Upper Volta[tiab] OR Burundi[tiab] OR Urundi[tiab] OR Cambodia[tiab] OR Khmer Republic[tiab] OR Kampuchea[tiab] OR Cameroon[tiab] OR Cameroons[tiab] OR Cameron[tiab] OR Camerons[tiab] OR Cape Verde[tiab] OR Central African Republic[tiab] OR Chad[tiab] OR Chile[tiab] OR China[tiab] OR Colombia[tiab] OR Comoros[tiab] OR Comoro Islands[tiab] OR Comores[tiab] OR Mayotte[tiab] OR Congo[tiab] OR Zaire[tiab] OR Costa Rica[tiab] OR Côte d'Ivoire[tiab] OR Ivory Coast[tiab] OR Croatia[tiab] OR Cuba[tiab] OR Cyprus[tiab] OR Czechoslovakia[tiab] OR Czech Republic[tiab] OR Slovakia[tiab] OR Slovak Republic[tiab] OR Djibouti[tiab] OR French Somaliland[tiab] OR Dominica[tiab] OR Dominican Republic[tiab] OR East

Timor[tiab] OR East Timur[tiab] OR Timor Leste[tiab] OR Ecuador[tiab] OR Egypt[tiab] OR United Arab Republic[tiab] OR El Salvador[tiab] OR Eritrea[tiab] OR Estonia[tiab] OR Ethiopia[tiab] OR Fiji[tiab] OR Gabon[tiab] OR Gabonese Republic[tiab] OR Gambia[tiab] OR Gaza[tiab] OR Georgia Republic[tiab] OR Georgian Republic[tiab] OR Ghana[tiab] OR Gold Coast[tiab] OR Greece[tiab] OR Grenada[tiab] OR Guatemala[tiab] OR Guinea[tiab] OR Guam[tiab] OR Guiana[tiab] OR Guyana[tiab] OR Haiti[tiab] OR Honduras[tiab] OR Hungary[tiab] OR India[tiab] OR Maldives[tiab] OR Indonesia[tiab] OR Iran[tiab] OR Iraq[tiab] OR Isle of Man[tiab] OR Jamaica[tiab] OR Jordan[tiab] OR Kazakhstan[tiab] OR Kazakh[tiab] OR Kenya[tiab] OR Kiribati[tiab] OR Korea[tiab] OR Kosovo[tiab] OR Kyrgyzstan[tiab] OR Kirghizia[tiab] OR Kyrgyz Republic[tiab] OR Kirghiz[tiab] OR Kirgizstan[tiab] OR "Lao PDR"[tiab] OR Laos[tiab] OR Latvia[tiab] OR Lebanon[tiab] OR Lesotho[tiab] OR Basutoland[tiab] OR Liberia[tiab] OR Libya[tiab] OR Lithuania[tiab])) OR (Macedonia[tiab] OR Madagascar[tiab] OR Malagasy Republic[tiab] OR Malaysia[tiab] OR Malaya[tiab] OR Malay[tiab] OR Sabah[tiab] OR Sarawak[tiab] OR Malawi[tiab] OR Nyasaland[tiab] OR Mali[tiab] OR Malta[tiab] OR Marshall Islands[tiab] OR Mauritania[tiab] OR Mauritius[tiab] OR Agalega Islands[tiab] OR "Melanesia"[tiab] OR Mexico[tiab] OR Micronesia[tiab] OR Middle East[tiab] OR Moldova[tiab] OR Moldovia[tiab] OR Moldovian[tiab] OR Mongolia[tiab] OR Montenegro[tiab] OR Morocco[tiab] OR Ifni[tiab] OR Mozambique[tiab] OR Myanmar[tiab] OR Myanma[tiab] OR Burma[tiab] OR Namibia[tiab] OR Nepal[tiab] OR Netherlands Antilles[tiab] OR New Caledonia[tiab] OR Nicaragua[tiab] OR Niger[tiab] OR Nigeria[tiab] OR Northern Mariana Islands[tiab] OR Oman[tiab] OR Muscat[tiab] OR Pakistan[tiab] OR Palau[tiab] OR Palestine[tiab] OR Panama[tiab] OR Paraguay[tiab] OR Peru[tiab] OR Philippines[tiab] OR Philipines[tiab] OR Phillipines[tiab] OR Phillippines[tiab] OR Poland[tiab] OR Portugal[tiab] OR Puerto Rico[tiab] OR Romania[tiab] OR Rumania[tiab] OR Roumania[tiab] OR Russia[tiab] OR Russian[tiab] OR Rwanda[tiab] OR Ruanda[tiab] OR Saint Kitts[tiab] OR St Kitts[tiab] OR Nevis[tiab] OR Saint Lucia[tiab] OR St Lucia[tiab] OR Saint Vincent[tiab] OR St Vincent[tiab] OR Grenadines[tiab] OR Samoa[tiab] OR Samoan Islands[tiab] OR Navigator Island[tiab] OR Navigator Islands[tiab] OR Sao Tome[tiab] OR Saudi Arabia[tiab] OR Senegal[tiab] OR Serbia[tiab] OR Montenegro[tiab] OR Seychelles[tiab] OR Sierra Leone[tiab] OR Slovenia[tiab] OR Sri Lanka[tiab] OR Ceylon[tiab] OR Solomon Islands[tiab] OR Somalia[tiab] OR Sudan[tiab] OR Suriname[tiab] OR Surinam[tiab] OR Swaziland[tiab] OR Syria[tiab] OR Syrian[tiab] OR Tajikistan[tiab] OR Tadzhikistan[tiab] OR Tadjikistan[tiab] OR Tadzhik[tiab] OR Tanzania[tiab] OR Thailand[tiab] OR Togo[tiab] OR Togolese Republic[tiab] OR Tonga[tiab] OR Trinidad[tiab] OR Tobago[tiab] OR Tunisia[tiab] OR Turkey[tiab] OR Turkmenistan[tiab] OR Turkmen[tiab] OR Tuvalu[tiab] OR Uganda[tiab] OR Ukraine[tiab] OR Uruguay[tiab] OR USSR[tiab] OR Soviet Union[tiab] OR Union of Soviet Socialist Republics[tiab] OR Uzbekistan[tiab] OR Uzbek OR Vanuatu[tiab] OR New Hebrides[tiab] OR Venezuela[tiab] OR Vietnam[tiab] OR Viet Nam[tiab] OR West Bank[tiab] OR Yemen[tiab] OR Yugoslavia[tiab] OR Zambia[tiab] OR Zimbabwe[tiab] OR Rhodesia[tiab])) OR (Developing Countries[Mesh] OR Africa[Mesh:noexp] OR Africa, Northern[Mesh:noexp] OR Africa South of the Sahara[Mesh:noexp] OR Africa, Central[Mesh:noexp] OR Africa, Eastern[Mesh:noexp] OR Africa, Southern[Mesh:noexp] OR Africa, Western[Mesh:noexp] OR Asia[Mesh:noexp] OR Asia, Central[Mesh:noexp] OR Asia, Southeastern[Mesh:noexp] OR Asia, Western[Mesh:noexp] OR Caribbean Region[Mesh:noexp] OR West Indies[Mesh:noexp] OR South America[Mesh:noexp] OR Latin America[Mesh:noexp] OR Central America[Mesh:noexp] OR "Atlantic Islands"[Mesh:noexp] OR "Commonwealth of Independent States"[Mesh:noexp] OR "Pacific Islands"[Mesh:noexp] OR "Indian Ocean Islands"[Mesh:noexp] OR "Europe, Eastern"[Mesh:noexp])) OR (Afghanistan[Mesh] OR Albania[Mesh] OR Algeria[Mesh] OR American Samoa[Mesh] OR Angola[Mesh] OR "Antigua and Barbuda"[Mesh] OR Argentina[Mesh] OR Armenia[Mesh] OR Azerbaijan[Mesh] OR Bahrain[Mesh] OR "Baltic States"[Mesh] OR Bangladesh[Mesh] OR Barbados[Mesh] OR Benin[Mesh] OR "Republic of Belarus"[Mesh] OR Belize[Mesh] OR Bhutan[Mesh] OR Bolivia[Mesh] OR Bosnia-Herzegovina[Mesh] OR Botswana[Mesh] OR Brazil[Mesh] OR Bulgaria[Mesh] OR Burkina Faso[Mesh] OR Burundi[Mesh] OR Cambodia[Mesh] OR Cameroon[Mesh] OR Cape Verde[Mesh] OR Central African Republic[Mesh] OR Chad[Mesh] OR Chile[Mesh] OR China[Mesh] OR Colombia[Mesh] OR Comoros[Mesh] OR Congo[Mesh] OR Costa Rica[Mesh] OR Côte d'Ivoire[Mesh] OR Croatia[Mesh] OR Cuba[Mesh] OR Cyprus[Mesh] OR Czechoslovakia[Mesh] OR Czech Republic[Mesh] OR Slovakia[Mesh] OR Djibouti[Mesh] OR "Democratic Republic of the Congo"[Mesh] OR "Democratic People's Republic of Korea"[Mesh] OR Dominica[Mesh] OR Dominican Republic[Mesh] OR East Timor[Mesh] OR Ecuador[Mesh] OR Egypt[Mesh] OR El Salvador[Mesh] OR Eritrea[Mesh] OR Estonia[Mesh] OR Ethiopia[Mesh] OR "Equatorial Guinea"[Mesh] OR Fiji[Mesh] OR "French Guiana"[Mesh] OR Gabon[Mesh] OR Gambia[Mesh] OR "Georgia (Republic)"[Mesh] OR Ghana[Mesh] OR Greece[Mesh] OR Grenada[Mesh] OR Guatemala[Mesh] OR Guinea[Mesh] OR Guinea-Bissau[Mesh] OR Guam[Mesh] OR Guyana[Mesh] OR Haiti[Mesh] OR Honduras[Mesh] OR Hungary[Mesh] OR "Independent State of Samoa"[Mesh] OR India[Mesh] OR Indonesia[Mesh] OR Iran[Mesh] OR Iraq[Mesh] OR Jamaica[Mesh] OR Jordan[Mesh] OR

Kazakhstan[Mesh] OR Kenya[Mesh] OR Korea[Mesh] OR Kyrgyzstan[Mesh] OR Laos[Mesh] OR Latvia[Mesh] OR Lebanon[Mesh] OR Lesotho[Mesh] OR Liberia[Mesh] OR Libya[Mesh] OR Lithuania[Mesh] OR "Macedonia (Republic)"[Mesh] OR Madagascar[Mesh])) OR (Malawi[Mesh] OR Malaysia[Mesh] OR Mali[Mesh] OR Malta[Mesh] OR Mauritania[Mesh] OR Mauritius[Mesh] OR "Melanesia"[Mesh] OR Mexico[Mesh] OR Micronesia[Mesh] OR Middle East[Mesh:noexp] OR Moldova[Mesh] OR Mongolia[Mesh] OR Montenegro[Mesh] OR Morocco[Mesh] OR Mozambique[Mesh] OR Myanmar[Mesh] OR Namibia[Mesh] OR Nepal[Mesh] OR Netherlands Antilles[Mesh] OR New Caledonia[Mesh] OR Nicaragua[Mesh] OR Niger[Mesh] OR Nigeria[Mesh] OR Oman[Mesh] OR Pakistan[Mesh] OR Palau[Mesh] OR Panama[Mesh] OR Papua New Guinea[Mesh] OR Paraguay[Mesh] OR Peru[Mesh] OR Philippines[Mesh] OR Poland[Mesh] OR Portugal[Mesh] OR Puerto Rico[Mesh] OR "Republic of Korea"[Mesh] OR Romania[Mesh] OR Russia[Mesh] OR "Russia (Pre-1917)"[Mesh] OR Rwanda[Mesh] OR "Saint Kitts and Nevis"[Mesh] OR Saint Lucia[Mesh] OR "Saint Vincent and the Grenadines"[Mesh] OR Samoa[Mesh] OR Saudi Arabia[Mesh] OR Senegal[Mesh] OR Serbia[Mesh] OR Montenegro[Mesh] OR Seychelles[Mesh] OR Sierra Leone[Mesh] OR Slovenia[Mesh] OR Sri Lanka[Mesh] OR Somalia[Mesh] OR South Africa[Mesh] OR Sudan[Mesh] OR Suriname[Mesh] OR Swaziland[Mesh] OR Syria[Mesh] OR Tajikistan[Mesh] OR Tanzania[Mesh] OR Thailand[Mesh] OR Togo[Mesh] OR Tonga[Mesh] OR "Trinidad and Tobago"[Mesh] OR Tunisia[Mesh] OR Turkey[Mesh] OR Turkmenistan[Mesh] OR Uganda[Mesh] OR Ukraine[Mesh] OR Uruguay[Mesh] OR USSR[Mesh] OR Uzbekistan[Mesh] OR Vanuatu[Mesh] OR Venezuela[Mesh] OR Vietnam[Mesh] OR Yemen[Mesh] OR Yugoslavia[Mesh] OR Zambia[Mesh] OR Zimbabwe[Mesh]))) AND (("Anti-Retroviral Agents"[Mesh] OR "Anti-Retroviral Agents"[Pharmacological Action] OR "ART"[tiab] OR "anti-retroviral agents"[tiab] OR "anti-retroviral treatment"[tiab] OR "anti-retroviral therapy"[tiab] OR "antiretroviral"[tiab] OR "Antiretroviral Therapy, Highly Active"[MeSH] OR "HAART"[tiab]) AND (HIV[MeSH] OR AIDS[MeSH] OR "HIV Infections"[MeSH] OR "Anti-Retroviral Agents"[MeSH] OR HIV[tw] OR HIV-1[tw] OR HIV-2[tw] OR Human immunodeficiency virus*[tw] OR Human Immunodeficiency Virus*[tw] OR AIDS[tw] OR Acquired Immunodeficiency Syndrome*[tw] OR "Acquired Immune Deficiency Syndrome"[tw] OR "Acquired Immune Deficiency Syndromes"[tw] OR "Acquired Immuno-Deficiency Syndrome"[tw] OR "Acquired Immuno-Deficiency Syndromes"[tw] OR "Acquired Immuno Deficiency Syndrome"[tw] OR "Acquired Immuno Deficiency Syndromes"[tw] OR "acquired immunologic deficiency syndrome"[tw]))) NOT (pediatrics[MESH] OR pediatrics[tiab] OR children[MeSH])

Table 6: Baseline Factors Adjusted for in Mortality Hazard Ratios*

Author, Year	Age	CD4 count	WHO Stage	Viral Load	BMI or Weight	Previous/ Current TB	Adh- erence	Hemo- globin (Anemia)	Additional Factors	Ref
**Africa**										
Abaasa, 2008	✔						✔		Education level	[21]
Alemu, 2010									-	[24]
Bastard, 2011		✔					✔	✔	-	[27]
Biadgilign, 2012	✔	✔	✔		✔				Functional status[a] education level, employment, religion, marital status	[28]
Bisson, 2008	✔	✔		✔				✔	-	[30]
Boyles, 2011	✔	✔				✔			Starting ART as inpatient, starting ART in pregnancy, pre-ART care > 6 months	[31]
Brinkhof, 2009	✔	✔	✔						Initial ART regimen, ART start year	[33]
Chalamilla, 2012		✔	✔		✔	✔		✔	ART regimen, patient referral site, calendar month, calendar year (age not significant in univariate analysis, so not included in multivariate)	[34]
Chen, 2008	✔		✔		✔				-	[35]
Chi, 2009	✔	✔	✔		✔		✔	✔	Adherence supporter, initial ART regimen	[36]
Chi, 2010	✔	✔	✔		✔	✔		✔	Serum creatinine, third ART drug; NRTI backbone	[37]
Chu, 2010									KS stage, bleomycin chemotherapy	[38]
Cornell, 2012	✔	✔	✔	✔	✔			✔	Cohort (site)	[40]
De Beaudrap, 2008									-	[42]
De Luca, 2012	✔	✔	✔	✔	✔		✔	✔	ART start year; 6-month post-initiation CD4 count & VL	[43]
Desilva, 2009	✔	✔				✔			Employment status	[45]
Fatti, 2010	✔	✔	✔						ART start year, province, facility level	[48]
Ford, 2010	✔	✔							Pregnant at initiation	[49]
Geng, 2010a	✔	✔							Blood pressure; central nervous system syndrome	[52]
Greig, 2012	✔				✔	✔			Severe immunosuppression, setting	[54]
Hawkins, 2011	✔	✔	✔		✔	✔		✔	Site, district, season, calendar year, antiretroviral regimen, hepatitis B and C status; alanine aminotransferase	[55]

Hermans, 2012	✔	✔							ART start year	[56]
Hoffman, 2011	✔	✔	✔	✔	✔	✔		✔	Cotrimoxazol use	[58]
Johannessen, 2008			✔		✔			✔	ART start year, total lymphocyte count, platelet count	[59]
Kassa, 2012	✔	✔	✔			✔			Functional status [a]	[61]
Kouanda, 2012	✔	✔	✔		✔				Profession, treatment regimen, ART start year, intensity of intervention	[64]
MacPherson, 2009	✔	✔	✔		✔				-	[66]
Mageda, 2012	✔	✔	✔		✔				Marital status, type of clinic, residence	[67]
Maman, 2012b	✔	✔	✔		✔				Updated CD4 cell count; time period of ART start	[69]
Maman, 2012c	✔		✔		✔				Chronic energy deficiency	[70]
Mutevedzi, 2011	✔	✔	✔	✔	✔	✔		✔	3 & 12 mo analyses: estimated glomerular filtration rate; 24 mo analysis: CD4 count at 6 & 12 months, VL at 12 months; all analyses: albumin	[79]
Negin, 2011	✔								-	[81]
Odafe, 2012	✔	✔	✔						Functional status, [a] type of facility	[83]
Ojikutu, 2008	✔	✔				✔			Employment status; oral candidiasis	[84]
Palombi, 2009	✔	✔		✔	✔		✔	✔	Model 1. Calendar year. (Model 2 also adjusted for WHO stage, which had 39% missing data)	[85]
Peterson, 2011	✔	✔	✔		✔			✔	ART start year, in cohort <12 months prior to ART initiation	[88]
Poka-Mayap, 2013	✔	✔	✔		✔	✔		✔	Urban/rural residency; platelet count; AZT-based regimen	[89]
Russell, 2010	✔	✔						✔	-	[91]
Sieleunou, 2009		✔	✔		✔			✔	(Age not significant in univariate analysis)	[93]
Somi, 2012	✔	✔	✔		✔				-	[95]
Toure, 2008	✔	✔	✔		✔		✔	✔	ART regimen, type of HIV seropositivity, type of care center	[99]
Wandeler, 2012	✔	✔	✔						ART start year, country, treatment regimen	[101]
Wubshet, 2012		✔	✔			✔			Functional status [a]	[103]
**Central Europe/Eastern Europe**										
Tsertsvadze, 2011		✔							AID defining illness, cirrhosis; (Age injection drug use, VL, presence of anti-Hepatitis C Virus antibodies and Hepatitis B antigens not significant in univariate analysis)	[15]
**Latin America**										
Wolff, 2010	✔	✔	✔	✔					Third drug (Efavirenz, nevirapine, Protease	[105]

									inhibitor), start year of ART	
**Asia**										
Alvarez-Uria, 2013	✔	✔							Disadvantaged community, Illiteracy, Homelessness, Living near a town, Poverty, Marital status	[106]
Argemi, 2012									-	[107]
Bastard, 2013		✔			✔				-	[108]
Chen, 2013	✔	✔							Mode of transmission (e.g. injection drug use), marital status	[110]
Fregonese, 2012	✔	✔	✔	✔				✔	(3-6 month analysis) -	[112]
	✔	✔		✔	✔			✔	(6+ month analysis) ART start year, CD4 count = increase at 6 months	
Rai, 2013	✔	✔					✔		Education, Household income, source of referral, opportunistic infections, distance to ART center	[115]
Tran, 2013, Vietnam	✔	✔	✔		✔	✔	✔		Initiation year, history of injection drug use, history of unsafe sex, baseline opportunistic infections, baseline cotrimoxazol use	[119]
Van Griensven, 2011		✔			✔			✔	(Age, WHO stage, ART start year were not significant in univariate analysis)	[120]
Zhang, 2009	✔	✔						✔	Marital status, infection route (sexual vs. injection drug use vs. plasma donation), alanine aminotransferase level, baseline symptoms category	[122]

a e.g. bedridden, ambulatory, working
*All studies in Model 1

Table 7: Final Meta-Analysis (Model 4) Inclusions and Exclusions of Studies with Mortality Data

1st author, year	Included	Excluded	Reason	HR*	95% CI
**Africa**					
Abaasa, 2008		✔	LTFU ≥15%	1.48	0.98-2.17
Ahonkhai, 2012		✔	No HR; Odds ratio only		
Alamo, 2012		✔	No HR; Crude measure only		
Alemu, 2010		✔	Moderate quality; LTFU ≥15%	1.22	0.58-2.56
Alibhai, 2010		✔	No HR; Crude measure only		
Balcha, 2010		✔	No HR; Crude measure only		
**Bastard, 2011**	✔		**High quality; LTFU <15%**	**2.13**	**1.19-3.85**
**Biadgilign, 2012**	✔		**High quality; LTFU <15%**	**0.86**	**0.5-1.47**
Birbeck, 2011		✔	No HR; Odds ratio only		
Bisson, 2008		✔	LTFU >15%	1.74	1.05-2.87
**Boyles, 2011**	✔		**High quality; LTFU <15%**	**0.83**	**0.60-1.15**
Brennan, 2013		✔	No HR; relative risk ratio only		
Brinkhof, 2009		✔	LTFU ≥15%	1.19	1.01-1.41
Chalamilla, 2012		✔	LTFU not reported	1.19	1.07-1.32
Chen, 2008		✔	LTFU ≥15%	1.70	1.35-2.15
Chi, 2009		✔	LTFU ≥15%	1.30	1.10-1.50
Chi, 2010		✔	LTFU ≥15%	1.82	1.47-2.26
**Chu, 2010**	✔		**High quality; LTFU <15%**	**0.98**	**0.73-1.31**
Cornell, 2009		✔	Cohort also represented in Cornell 2012[a]		
**Cornell, 2012**	✔		**High quality; LTFU <15%**	**1.10**<br>**1.36**<br>**1.39**<br>**1.35**	**0.93-1.31**<br>**1.05-1.78**<br>**0.94-2.06**<br>**0.76-2.38**
Dalal, 2008		✔	No HR; Crude measure only		
**De Beaudrap, 2008**	✔	✔	**0-6 month HR: High quality; LTFU <15%**<br>7-84 month HR: Excluded, LTFU ≥15%	**0.97**<br>**1.45**	**0.47-2.00**<br>**0.86-2.38**
**De Luca, 2012**	✔		**High quality; LTFU <15%**	**1.76**	**1.11-2.80**
Deribe, 2013		✔	No HR; Odds ratio only		
**Desilva, 2009**	✔		**High quality; LTFU <15%**	**1.76**	**1.14-2.72**
Ekouevi, 2010		✔	Provides combination only of mortality plus LTFU		
Evans, 2012		✔	Cohort included in Cornell 2012 [a]		
**Fatti, 2010**	✔		**High quality; LTFU <15%**	**1.14**	**1.00-1.30**

Ford, 2010		✔	LTFU ≥15%	0.90	0.34-2.38
Fox, 2010		✔	Cohort also represented in Cornell 2012 [a]		
Fox, 2012		✔	Cohort also represented in Cornell 2012 [a]		
Franke, 2011		✔	Entire sample had current tuberculosis		
Geng, 2010a		✔	LTFU ≥15%	1.54	0.53-4.12
Geng, 2010b		✔	Cohort also represented in Geng 2010a [a]; of 3 HRs reported with 3 analyses, chose to include the one in the middle		
Greig, 2012		✔	LTFU % not reported	1.20<br>1.02	1.01-1.41<br>0.84-1.22
Hawkins, 2011		✔	LTFU ≥15%	1.19	1.05-1.30
**Hermans, 2012**	✔		**High quality; LTFU <15%**	**1.41**	**1.12-1.77**
Hoffmann, 2010		✔	Cohort also represented in Hoffmann 2011[a]; smaller n		
Hoffmann, 2011		✔	LTFU ≥15%	1.20	1.00-1.30
**Johannessen, 2008**	✔		**High quality; LTFU <15%**	**1.60**	**1.00-2.57**
Karstaedt, 2012		✔	No HR; Crude measure only		
**Kassa, 2012**	✔		**High quality; LTFU <15%**	**1.07**	**0.84-1.36**
Kebebew, 2012		✔	No HR; Crude measure only		
Kipp, 2012		✔	No HR; Crude measure only		
**Kouanda, 2012**	✔		**High quality; LTFU <15%**	**1.33**	**1.05-1.68**
Lowrance, 2009		✔	No HR; Odds ratio only		
**MacPherson, 2009**	✔		**High quality; LTFU <15%**	**1.63**	**1.12-2.36**
Mageda, 2012		✔	LTFU not reported	4.71	2.00-11.05
Maman, 2012a		✔	No HR; Crude measure only		
Maman, 2012b		✔	LTFU not reported		
**Maman, 2012c**	✔		**High quality; LTFU <15%**	**1.36**	**0.93-2.00**
Maskew, 2012		✔	Cohort also represented in Cornell 2012[a]		
Maskew, 2013		✔	Cohort also represented in Cornell 2012[a]		
Massaquoi, 2009		✔	No HR; Crude measure only		
Moore, 2011		✔	No HR; Crude measure only		
Mossdorf, 2011		✔	Provides combination only of mortality plus LTFU		
Mosha, 2013		✔	No HR; Crude measure only		
Mujugira, 2009		✔	Entire sample was only sickest patients (CD4 <50)		
Murphy, 2010		✔	No HR; Odds ratio only		
Mutevedzi, 2010		✔	Cohort also represented in Mutevedzi 2011		

**Mutevedzi, 2011**	✔		**High quality; LTFU <15%**	**1.64**<br>**1.84**<br>**1.40**<br>**1.33**<br>**1.95**	**1.32-2.03**<br>**1.06-3.17**<br>**1.09-1.80**<br>**0.73-2.41**<br>**1.46-2.57**
Mzileni, 2008		✔	No HR; Crude measure only		
Negin, 2011		✔	LTFU ≥15%	1.92	1.67-2.17
Nglazi, 2011		✔	Cohort also represented in Cornell 2012[a]		
Odafe, 2012		✔	LTFU ≥15%	1.29	0.89-1.86
**Ojikutu, 2008**	✔		**High quality; LTFU <15%**	**1.33**	**0.73-2.50**
**Palombi, 2009**	✔		**High quality; LTFU <15%;** Model 1 used	**1.96**	**1.59-2.50**
Palombi, 2010		✔	Cohort also represented in Palombi 2009 [a]	-	-
Peltzer, 2011		✔	No HR; relative risk only		
**Peterson, 2011**	✔		**High quality; LTFU <15%**	**4.9**<br>**2.50**	**2.5-10.8**<br>**1.20-5.60**
Poka-Mayap, 2013		✔	LTFU ≥15%	2.15	1.34-3.45
Rougemont, 2009		✔	No HR; Odds ratio only		
**Russell, 2010**	✔		**High quality; LTFU <15%**	**1.15**	**0.83-1.59**
Schoni-Affolter, 2011		✔	No HR; Crude measure only		
**Sieleunou, 2009**	✔		**High quality; LTFU <15%**	**1.73**	**1.37-2.19**
Siika, 2010		✔	Case-control study		
Somi, 2012	✔		LTFU ≥15%	1.70	1.61-1.79
Steele, 2011		✔	No HR: relative risk ratio only		
Sunpath, 2012		✔	Entire sample had current tuberculosis		
Taylor-Smith, 2010		✔	No HR: relative risk ratio only		
**Toure, 2008**	✔		**High quality; LTFU <15%**	**1.52**	**1.29-1.80**
Van Cutsem, 2011		✔	Cohort also represented in Cornell 2012[a]		
Wandeler, 2012		✔	LTFU ≥15%	1.45	1.25-1.70
Weigel, 2011		✔	Cross-sectional study design; Odds ratio only		
Wubshet, 2012		✔	LTFU ≥15%	3.26	2.19-4.88
Zachariah, 2009		✔	No HR; Odds ratio only		
**Central Asia/Eastern Europe**					
**Tsertsvadze, 2011**	✔		**High quality; LTFU <15%**	**1.96**	**1.19-3.24**
**Latin America**					
**Wolff, 2010**	✔		**High quality; LTFU <15%**	**1.24**	**0.93-1.64**

**Asia**					
Alvarez-Uria, 2013	✔		LTFU ≥15%	1.53	1.21-1.92
**Argemi, 2012**	✔		**High quality; LTFU <15%**	**1.28**	**0.88-1.88**
**Bastard, 2013**	✔		**High quality; LTFU <15%**	**0.84**<br>**5.89**	**0.49-1.45**<br>**2.27-14.29**
Bhowmik, 2012		✔	No HR; Crude measure only		
**Chen, 2013**	✔		**High quality; LTFU <15%**	**2.10**	**1.20-3.50**
Dou, 2011		✔	Cohort also represented in Zhang 2009 [a]		
**Fregonese, 2012**	✔		**High quality; LTFU <15%**	**1.50**<br>**2.40**	**0.70-3.10**<br>**1.20-4.80**
Kumarasamy, 2008		✔	No HR; Crude measure only		
Limmahakhun, 2012		✔	No HR; Crude measure only		
**Rai, 2013**	✔		**High quality; LTFU <15%**	**2.80**	**1.60-4.90**
Sabapathy, 2012		✔	Provides combination only of mortality plus LTFU		
Susaengrat, 2011		✔	No HR; Crude measure only		
Thai, 2009		✔	Provides combination only of mortality plus LTFU		
Tran, 2013		✔	LTFU ≥15%	1.85	1.18-2.94
**van Griensven, 2011**	✔		**High quality; LTFU <15%**	**2.08**<br>**1.72**	**1.35-3.23**<br>**1.01-2.94**
Zhang, 2008		✔	Cohort also represented in Zhang 2009 [a]		
**Zhang, 2009**	✔		**High quality; LTFU <15%**	**1.40**	**1.30-1.50**
Zhang, 2012		✔	Cohort also represented in Zhang 2009 [a]		
Brinkhof, 2008		✔	Cohort also represented in Brinkhof 2009 [a]		
Wandel, 2008		✔	HRs reported incompletely; not useable in MA		

*HRs listed here were considered in sensitivity analyses; **Bold** studies were included in the final model; For studies that reported HR females vs. males, the reciprocals were calculated and are reported in this table

[a] Overlap of cohorts determined through review of description in studies' methods sections, e.g. follow-up months/years, sample size, name of cohort, sites and settings, and co-authorship

Figure 3: Funnel Plot of Included Studies (Model 4)

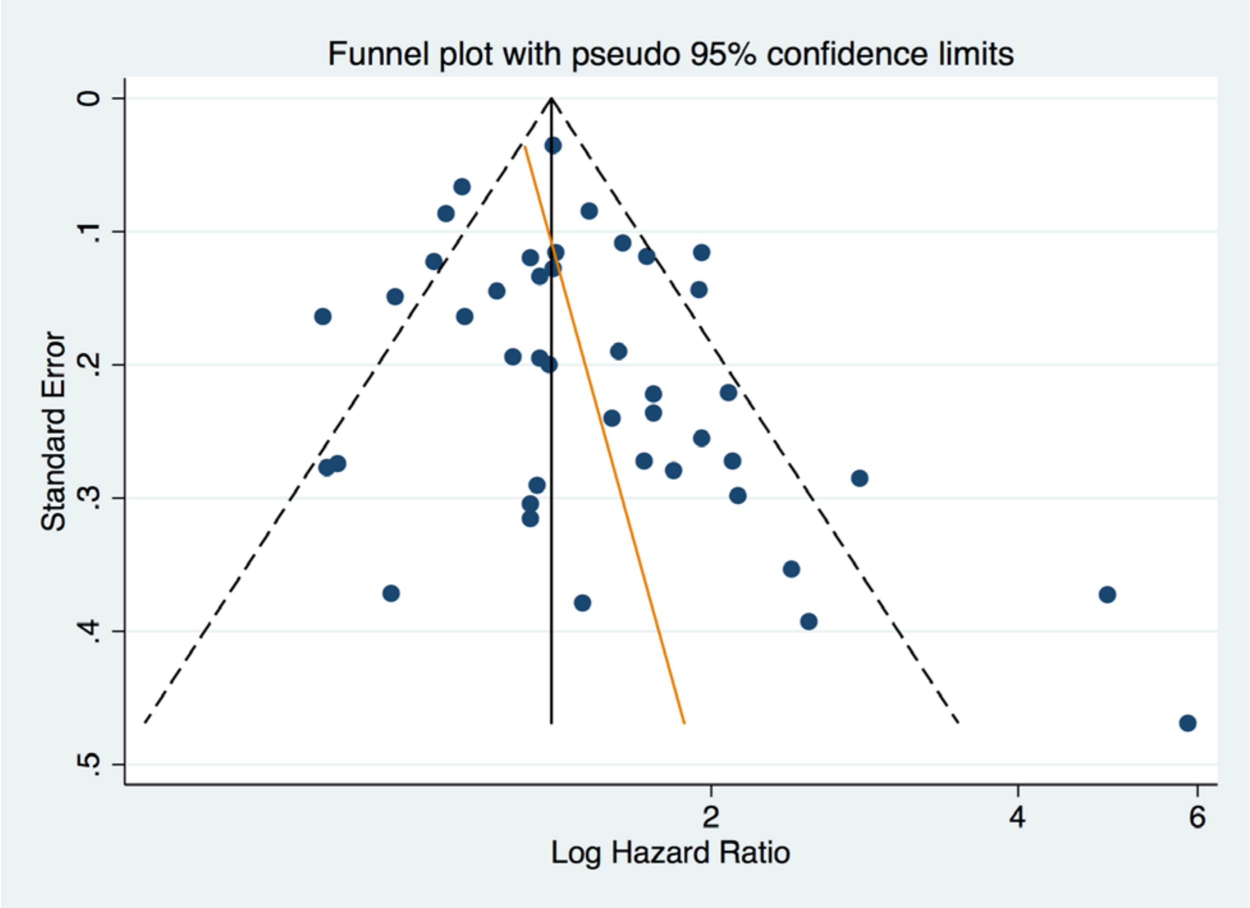

Supplement: Marked sex differences in all-cause mortality on antiretroviral therapy in low- and middle-income countries: a systematic review and meta-analysis [file JIAS-19-21106-s001.pdf]
